# Non-clinical interventions to reduce unnecessary caesarean section targeted at organisations, facilities and systems: Systematic review of qualitative studies

**DOI:** 10.1371/journal.pone.0203274

**Published:** 2018-09-04

**Authors:** Carol Kingdon, Soo Downe, Ana Pilar Betran

**Affiliations:** 1 Department of Community Health and Midwifery, University of Central Lancashire, Preston, Lancashire, United Kingdom; 2 Department of Reproductive Health and Research, World Health Organization (WHO), Geneva, Switzerland; Aga Khan University, KENYA

## Abstract

**Objective:**

When medically indicated, caesarean section can prevent deaths and other serious complications in mothers and babies. Lack of access to caesarean section may result in increased maternal and perinatal mortality and morbidity. However, rising caesarean section rates globally suggest overuse in healthy women and babies, with consequent iatrogenic damage for women and babies, and adverse impacts on the sustainability of maternity care provision. To date, interventions to ensure that caesarean section is appropriately used have not reversed the upward trend in rates. Qualitative evidence has the potential to explain why and how interventions may or may not work in specific contexts. We aimed to establish stakeholders’ views on the barriers and facilitators to non-clinical interventions targeted at organizations, facilities and systems, to reduce unnecessary caesarean section.

**Methods:**

We undertook a systematic qualitative evidence synthesis using a five-stage modified, meta-ethnography approach. We searched MEDLINE, CINAHL, PsychINFO, EMBASE and grey literature databases (Global Index Medicus, POPLINE, AJOL) using pre-defined terms. Inclusion criteria were qualitative and mixed-method studies, investigating any non-clinical intervention to reduce caesarean section, in any setting and language, published after 1984. Study quality was assessed prior to data extraction. Interpretive thematic synthesis was undertaken using a barriers and facilitators lens. Confidence in the resulting Summaries of Findings was assessed using GRADE-CERQual.

**Results:**

8,219 studies were identified. 25 studies were included, from 17 countries, published between 1993–2016, encompassing the views of over 1,565 stakeholders. Nineteen Summary of Findings statements were derived. They mapped onto three distinct themes:

Health system, organizational and structural factors (6 SoFs); Human and cultural factors (7 SoFs); and Mechanisms of effect to achieve change factors (6 SoFs). The synthesis showed how inter- and intra-system power differentials, and stakeholder commitment, exert strong mechanisms of effect on caesarean section rates, independent of the theoretical efficacy of specific interventions to reduce them.

**Conclusions:**

Non-clinical interventions to reduce caesarean section are strongly mediated by organisational power differentials and stakeholder commitment. Barriers may be greatest where implementation plans contradict system and cultural norms.

**Protocol registration:**

PROSPERO: CRD42017059456

## Introduction

Although, over recent decades, maternity care provision has resulted in improvements in maternal and infant health, there is increasing evidence of the phenomenon that has been characterised *as ‘Too much*, *too soon*, *too little*, *too late’*. [1,2] This describes the simultaneous over and underuse of interventions in pregnancy, labour and birth. Caesarean section epitomizes this situation, with substantial inequalities in caesarean section rates within and between countries. [1–3] At the same time as lack of access to caesarean section can result in increased maternal and perinatal mortality and morbidity, the global rise in caesarean section rates is associated with overuse in healthy women and babies, with consequent iatrogenic damage, and with adverse impacts on the sustainability of maternity care provision. [3–5]

Latest estimates show that rates are highest in middle-income countries and rising in most low-income countries. From 1990 to 2014, on average, caesarean section rates increased from 22.8% to 42.2% in Latin American and the Caribbean, 18.5% to 32.6% in Oceania, 22.3% to 32.3% in North America, 11.2% to 25% in Europe, 4.4% to 19.5% in Asia, and 2.9% to 7.4% in Africa. [6] In view of this unprecedented rise, in 2015, the World Health Organization (WHO) published a Statement on caesarean section declaring that caesarean section rates higher than 10% are not associated with reductions in maternal and newborn mortality rates, and, as for any surgical procedure, a caesarean section can result in complications, disability or death, particularly in settings that lack the facilities and/or capacity to properly conduct safe surgery. [7] Around the same time, the United Nation’s (UN) Sustainable Development Goals, [8] and calls for Right Care for health, [9] for every woman, every child, everywhere, [10] emerged as global health priorities. However, a reduction in the rate of increase in caesarean section has not yet followed these strategic intentions, with the additional hurdle that little is known about to how tackle the paradoxical over and underuse to achieve optimal caesarean section rates. [2] This is possibly because the reasons for excessive use of caesarean section are complex, and include non-clinical factors (such as maternal or clinician convenience, financial incentives, fear of litigation or social demands). [11]

In addition to the clinical and psychosocial factors that are known to affect caesarean section rates, health system, facility management and organizational factors are important aggregate-level determinants of caesarean section use. [12] Little is known about the influence of these agents on childbirth interventions, or about how these factors modulate the effectiveness of interventions to reduce caesarean section rates that are targeted at this level of the maternity care system. We present a qualitative evidence synthesis that aimed to add new insights into what stakeholders say are the barriers and facilitators to the implementation of non-clinical interventions to reduce unnecessary caesarean section targeted at organizations, facilities and systems (OFS).

## Materials and methods

We used a modified meta-ethnography methodological approach. [13] (S1 Table). In our protocol [14] (S1 Text) we specified six objectives relating to six kinds of interventions targeted at OFS (replicating the categorization used in the Cochrane Review of non-clinical interventions to reduce unnecessary caesarean section) [11,15]. These interventions were; different types of nurse/midwife and physician staffing models; changes in the physical environment of labour; predetermined caesarean section rates set at physician-, hospital- or regional-level; financial strategies; legal liability strategies; and organisational culture.

### Search strategy and selection criteria

Inclusion criteria were pre-specified as: qualitative or mixed-method studies reporting stakeholder views, undertaken in any setting where a non-clinical intervention to reduce unnecessary caesarean section targeted at OFS had been investigated or developed, published in any language, for which a full manuscript was available. Stakeholders could be anyone whose view was sought on an intervention. We surmised that stakeholders could include policy makers, healthcare managers, health professionals, women and families, but stated in our protocol that the category would be post-defined, depending on the nature of the included studies. We predefined an intervention as anything considered by the study authors as an intervention undertaken with the aim of reducing caesarean section, that was different to usual care. We, excluded clinical interventions. [14]

We searched CINAHL, MEDLINE, PsychINFO, EMBASE, Global Index Medicus, POPLINE and African Journals Online using MeSH and free-text terms combining up to four components: stakeholder populations; interventions of interest; caesarean section; and qualitative methods. Search strategies were informed by preliminary scoping searches, existing quantitative reviews of interventions to reduce caesarean section, [15–17], guidelines developed by the Cochrane Qualitative Research Methods Group, [18,19] and papers detailing strategies for optimising the identification of qualitative studies. [20–23] (S2 Text) A date restriction (1st January 1985 to date of last search: 22nd March 2017) was imposed to identify studies published since the WHO [24] consensus statement on caesarean section. We imposed no language or geographic restrictions. Back-chaining and forward checking of reference lists was undertaken. Key articles cited by multiple authors (citation pearls) were checked on Google Scholar. The authors of relevant published protocols were contacted. [25,26]

Records of included studies at the abstract stage were collated into one database and duplicates removed. Two review authors (CK,SD) independently assessed each abstract and full text to determine eligibility for inclusion against *a priori* inclusion and exclusion criteria. Three papers required translation and were found to be eligible for inclusion. [27–29] The view of the third author (APB) was sought before agreeing on the final list of included studies. Two studies quality assessed as C-D were excluded from the main analysis based on sampling decisions that prioritised geographical spread, and excluded lower quality studies if they were based in locations where sufficient good quality studies were already included. [30,31] These two studies and two others [32,33] investigating organisational culture in general (rather than a targeted cultural change) were used in a confirmatory capacity to test the fit of the line of argument that emerged from the study.

### Data analysis

The analytic process followed a broad Qualitative Evidence Synthesis (QES) approach. Following the principles of meta-ethnography [13] data extraction and analyses were undertaken simultaneously. We did this in five stages:

1. **Familiarisation and quality assessment** of individual studies was independently undertaken by two authors (CK,SD) using the criteria described by Walsh [34] and the A-D grading of Downe. [35]
2. **Data extraction** whereby the characteristics of included studies, verbatim text (participant quotes) and author interpretation (themes, theories and metaphors) were entered into a form designed specifically for the purposes of the review, beginning with the earliest paper. [36]
3. **Coding** with codes constructed using extracted data from the first paper and then comparing it with the findings from another until all extracted data from all included studies were coded into initial concepts.
4. **Interpretative synthesis** was the process of grouping initial concepts into emergent themes (also termed Summary of Findings (SoFs) in QES analysis), first by looking for what was similar between the studies we had already looked at, and the one currently under review (termed ‘reciprocal analysis’), and then by looking for what might be different between the previous analysis and the paper currently under review (termed ‘refutational analysis’). This process resulted in a set of Summaries of Findings (SoFs) that explained a range of barriers and facilitators to change. The SoFs were then synthesised into final themes, and these were translated into a Line of Argument statement.
5. **GRADE-CERQual** is an approach to assess the confidence in qualitative evidence synthesis findings. [37,38] Assessment was undertaken at the level of the SoFs, with each one assessed for four criteria: methodological quality of studies underpinning the SoF, coherence across those studies, relevance to the review question, and adequacy. Based on the GRADE approach, each SoFs was initially given a high confidence rating, and then downgraded to moderate, low or very low confidence depending on the degree to which each of these criteria were not met. (S2 Table).

#### Reflexive statement

Reflexive accounting allows the reader of the final research product to assess the degree to which the prior views and experiences of the researcher may have influenced the design, data collection and data interpretation of the study or in this case, the synthesis of the findings of multiple studies. This review was conceived with an informed knowledge of caesarean section and a degree of professional distance, which arguably limited bias based on the teams own experiences. APB is a medical officer with over 15 years of experience in maternal and perinatal health research and public health in general, and caesarean section in particular. CK, a medical sociologist, came to the project with prior beliefs about the complexity and interdependency of social factors driving caesarean section rates, principally informed by undertaking earlier primary research with women and health professionals in the UK. SD, a Professor of Midwifery, believed that maternity care organisations are complex adaptive systems, and that the organisational ethos can exert either toxic or enhancing effects that have real consequences for staff morale, engagement, attitudes, behaviours and performance.

## Results

**Fig 1:**
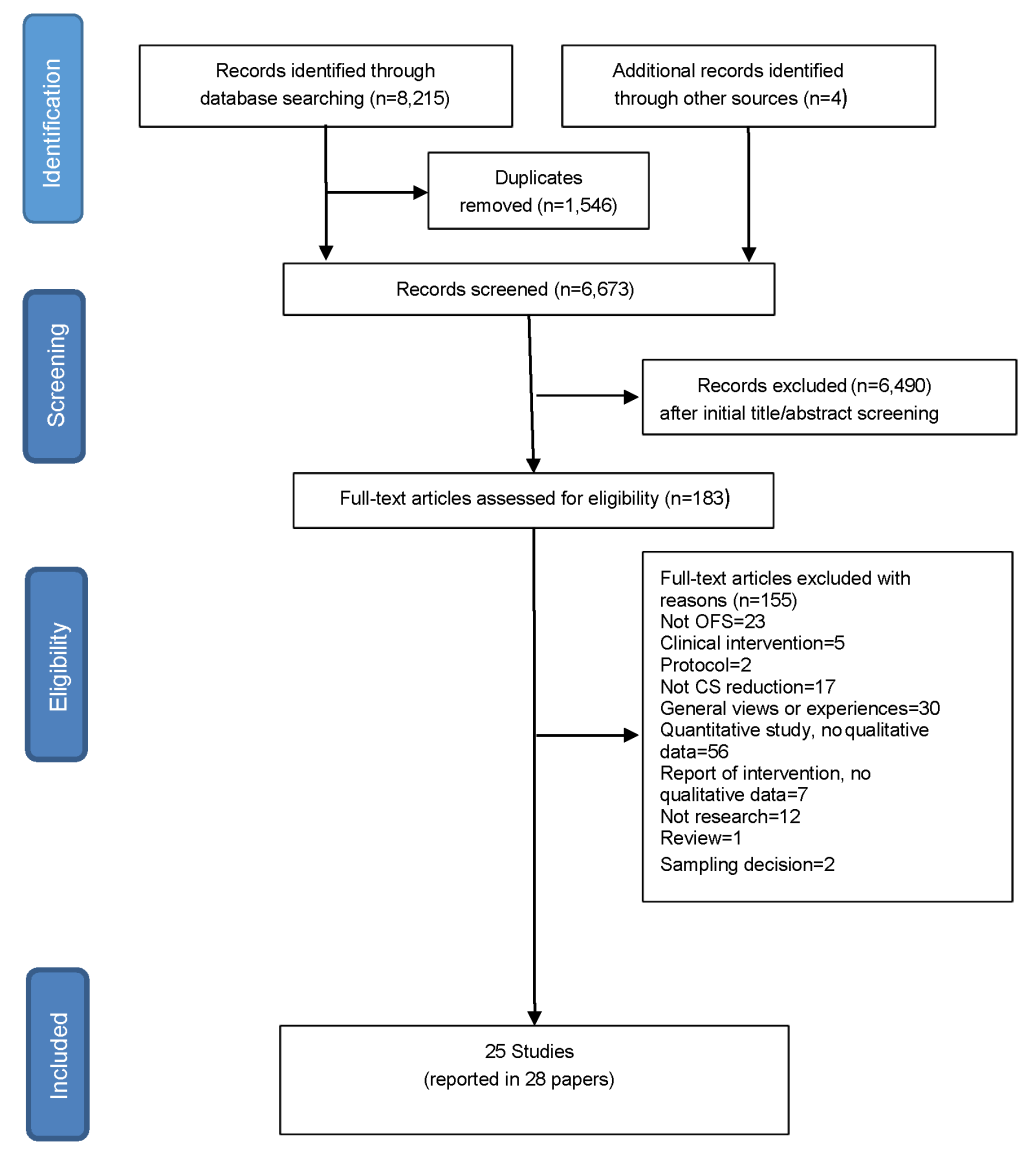
PRISMA diagram.

Twenty-five studies (reported in 28 papers) were included, from 17 countries, published between 1993 and 2016. Sample sizes ranged from 10 to 336 participants, and the views of over 1,565 stakeholders were included. [27–29,36,39–62] Stakeholders were policy makers, managers, health professionals, women, family members and community representatives. The database searches identified 8,215 studies; from CINAHL (n = 2,225), MEDLINE (n = 644), PsychINFO (n = 330), EMBASE (n = 958), Popline (n = 1,950), Global Index Medicus (n = 1,608) and African Journals Online (n = 500). Four further studies were identified by key informants and through back-chaining reference lists. [27,29,40,61] (Fig 1) Nineteen studies were graded A or B for quality. Five were graded C, and one D. Of the 25 studies, nine were from high-income countries, five from Africa, four from Latin America, three from China, two from Iran, one from Bangladesh and one from Lebanon. Table 1 describes the characteristics of the included studies, the type of intervention used, and the quality assessment.

**Table 1:** Characteristics of included studies and quality assessment.

Author	Aim	Country (Region)	Resource	Setting	Number of participants	Type of participants	Method	Quality Assessment
Binfa (2016)	To explore professionals' perceptions (obstetricians and midwives), as well as consumers' perceptions of this humanised assistance during labour and childbirth	Chile (Americas)	Middle	Rural and urban	96	Women, midwives and obstetricians	Focus groups	B
Kennedy (2016)	To investigate facilitators and barriers to the achievement of primary vaginal birth in first-time mothers in hospital settings, in light of growing interest in preventing primary caesarean deliveries	USA (Americas)	High	Urban	103	Caregivers/administrators and first-time mothers	Individual or small group interviews	B+
Lange (2016)	To capture pregnant women's experiences of quality of care, including the related costs and any financial barriers, when delivering in referral hospitals after the implementation of the user fee removal policies	Benin (African)	Low	Rural and urban	62	Women	Semi-structured Interviews and observations	A
Rishworth (2016)	To explore women’s experiences of caesarean birth in the context of Ghana’s maternal exemption policy	Ghana (African)	Middle	Rural	170	Women	Focus group discussions and in-depth interviews	A-
Witter (2016)	To document the costs and impacts of obstetric fee removal and reduction policies in a holistic way	Benin, Burkina Faso, Mali and Morocco (African)	Low	Rural and urban	336	Key informants	Interviews and observations	B
Janani (2015)	To explore challenges in implementing the PBP from perspective of midwives and obstetricians that provide maternity care	Iran (Eastern Mediterranean)	Middle	Urban	38	Obstetricians and midwives	Focus groups and semi-structured interviews	B-
Marshall (2015)	To evaluate the ‘Focus on Normal Birth and Reducing Caesarean section Rates’ programme	UK (European)	High	Rural and urban	30	Midwifery managers, lead Obstetricians, organisational development leads, clinical midwives and service users	Semi-structured interviews	A-
Colomar (2014)	To assess physicians’ and obstetric decision-makers’ opinions of the determinants of the high rate of caesarean births in Nicaragua as well as possible barriers to and facilitators of optimal caesarean birth rates	Nicaragua (Americas)	Middle	Unclear	17	Doctors and obstetric decision makers	Focus Groups	A
Hunter (2014, 2010a,2010b)	To explore how the All Wales Clinical Pathway for normal labour was developed and used in real life settings and evaluate its implementation from the perspectives of all key players: midwives, doctors, mothers and midwifery managers	UK (European)	High	Rural and urban	52	Midwives, midwifery managers, and doctors (obstetricians and GPs)	Observation, focus groups and interviews	A-
Dunn (2013)	To reduce high rates of ERCS < 39 weeks across the Eastern Ontario region	Canada (Americas)	High	Unclear	>9	Nursing Directors and Mangers	Key informant interviews	C
Cheyne (2013)	To explore and explain the ways in which the Keeping Childbirth Natural and Dynamic (KCND) programme worked or did not work in different maternity care contexts	UK (European)	High	Rural and urban	73	Health Professionals	Semi-structured interviews and focus groups	B+
Binfa (2013)	To explore the perception of this humanised attention during labour and delivery by both the professional staff (obstetricians and midwives) and consumers	Chile (Americas)	Middle	Urban	>8	Women, health professionals and Directors	Focus groups and in-depth interviews	B
Zhu (2013)	To explore factors influencing rates of caesarean section in China from organisational perspective	China (Western Pacific)	Middle	Urban	10	Policy makers and health managers	Focus group discussions	C
Huang (2012)	To investigate that NCMS may provide service users and providers with financial incentives to select caesarean section	China (Western Pacific)	Middle	Rural	>20	Health managers, providers and health service users	Focus group discussions and in-depth interviews	B-
Mbaye (2011)	To analyse the main reasons for high hospital caesarean section rates (i.e. above the national average) based on three cases of exemption from payment.	Senegal (African)	Low	Urban and rural	68	Medical and midwifery staff, administrators, service users, central-level managers/decision makers	Semi-structured interviews, focus group discussions, observation and document analysis	B-
Yazdizadeh (2011)	To identify barriers of reduce the caesarean section rate in Iran, as perceived by obstetricians and midwives as the main behavioural change target groups	Iran (Eastern Mediterranean)	Middle	Urban	26	Hospital directors, obstetricians and midwives	In-depth interviews	A-
Behruzi (2010)	To explore the Japanese birthing experience in hospitals that had implemented strategies aimed at reducing caesarean section and identified the humanization of birth as a priority goal	Japan (Western Pacific)	High	Urban	44	Midwives, Doctors and women	Observation, focus groups, informal and semi-structured interviews	A-
Liu (2010)	To explore factors affecting continuing increasing in caesarean section rate in rural area of China	China	Middle	Rural	82	Managers, obstetricians, women and family members	Interviews and focus groups	C
Schmidt (2010)	To assess early implementation of voucher scheme as demand side financing instruments for health care	Bangladesh (South-East Asia)	Low	Unclear	Unclear	Women, beneficiaries, service providers and Government officials	Key informant in-depth interviews and focus group discussions	D
Witter (2009, 2008)	To explore the views of the community and those with national, regional and district responsibility for the free delivery policy	Senegal (African)	Low	Urban and rural	160	Community representatives and key informants	In-depth interviews and focus groups	C+
Kabakian-Khasholian (2007)	To explore the potential for introducing a policy to reduce the CS rate in Lebanon	Lebanon (Eastern Mediterranean)	Middle	Unclear	66	Obstetricians, midwives, women who had a CS, hospital directors, insurance bodies, ministries, and media representatives	Semi-structured interviews and group discussion	B+
Shelp (2004)	To explore women’s views and experiences of the Somali Doula Initiative	USA (Americas)	High	Urban	60	Nurses and women	Surveys with free-text qualitative responses	C-
OWHC (2000)	To identify the critical factors associated with low caesarean section rates (policies, approaches, programs and services) at four of the best practice hospitals in Ontario	Canada (Americas)	High	Urban	> 4	Maternity care staff	Staff poll including qualitative responses	B-
Campero (1998)	To evaluate the effects of the provision of social support (doula) to first-time mothers during labour and childbirth	Mexico (Americas)	Middle	Urban	16	Women	In-depth interviews	A-
Sakala (1993)	To explore how midwives and out-of-hospital settings reduce unnecessary caesarean sections	USA (Americas)	High	Urban	15	Midwives	Semi-structured interviews	B-

The studies investigated stakeholder views of different types of midwife staffing models [36,50,52–54]; financial strategies [28,29,43–44,48,58–60]; and organisational culture [27,40,42,46,47,49,51,55–57,61,62]. We also included two studies of social (doula) support during labour [39,41], with the decision for inclusion made by consensus, because of their positive effect on caesarean section rate reduction in the associated Cochrane review of effectiveness studies. [15] We identified no studies specifically investigating views of legal liability interventions, changes to the physical environment, or interventions where predetermined caesarean section rates were set at physician-, hospital- or regional-level, although general views on these issues were reported in the context of particular staffing models and/or organisational culture.

Table 2 reports the SoFss table for this review, along with the CERQual ratings for each SoFs [37,38].

**Table 2:** Summary of findings and CERQual ratings.

Review finding	Contributing studies	*Assessment confidence in the evidence*	*Explanation of confidence in the evidence assessment*
**Summary theme 1. Health system, organizational and structural factors**			
**Professional power, roles and relationships:** Where interventions challenged the balance of power between professionals, concerns within and between professional groups in practice were widespread. Stakeholders included obstetricians, midwives, family doctors and women. In organisations implementing MLC programmes there was dissatisfaction from doctors who felt their professional identity and the safety of women was compromised by relinquishing lead professional responsibility to midwives. There was some evidence that financial strategies to reduce costs for service users might enable midwives/local skilled birth attendants to refer women to facilities/obstetricians for CS more freely.	42,46,47,49,50,52–54,57,58, 60,61,62	High confidence	11 studies with no or minor methodological limitations. Thick data from HICs and MICs with high CS rates. Thin data from LIC resource settings. High coherence.
**Fee exemption/reduction policies as mediators of access to necessary and unnecessary CS:** Across a number of studies, fee reduction policies were associated with a variable effect on appropriate use of CS dependent upon local philosophies of maternity care; inter-professional and inter-personal relationships; staff motivation to work with women or with the organisation, or simply for an income; and the expectations and demands of local women, families and communities. The unintended consequences of an increase in CS subsequent to reducing fees included longer-term iatrogenic damage to women’s health that is not covered by fee exemption.	28,43,44,58,59,60	Moderate confidence	Moderate confidence in LIC and MIC settings where fee exemption or reduction polices exist. 6 studies with no to major methodological limitations. All studies from LICs. Some thick data. Moderate coherence.
**Health insurance reform as a mediator of access to necessary and unnecessary CS:** Implementation of strategies to limit indications for CS accepted by insurance companies in Iran were met with scepticism about the power of insurance companies, concerns women who need a CS may no longer get one, and an increase in misreporting of indications for CS to satisfy amended insurance criteria. Insurance reform in China was not believed to be as influential on CS rates as women’s views of the advantages of CS.	27,29,47, 48	Very low confidence	4 studies with no to moderate methodological limitations. Major concerns about adequacy of data (thickness and spread). Too few studies contributed to this review finding to assess coherence.
**Birth environment, efficiency concerns and organisational logistics:** Only one included studied from the USA reported midwives’ views and experiences of birth in a home setting on the periphery (referring in if necessary) of birth in an organisation or facility, within a wider healthcare system. This study highlighted the absence of restrictions on women’s movements, environmental comforts, and time-limits evident in institutional settings. In the other studies contributing to this review finding a lack of time, space and facilities required for labour and normal birth were widely reported across resource contexts, as was access to operating theatres as a factor in clinical decision-making. In HICs where organisations had made changes to improve the birth environment and promote normal birth maintaining them was reported as a challenge (i.e. beds moved back in, resources for non-pharmacological forms of pain relief not prioritised). Insufficient space, insufficient staffing, lack of bathtubs, midwifery care not available for some women, and nutrition policies were commonly noted barriers. In MICs concerns were reported that delivery rooms were shared with other women (limiting presence of partner, family or other labour support companion), had inadequate facilities (lack of lighting, toilets, showers or baths, air-conditioning), or had been changed into operating theatres to accommodate rising numbers of CSs.	28,36,39, 40–42,46, 47,49, 51,55–58,61,62	High confidence	16 studies, most with minor methodological limitations. Thick data from 5 geographical regions and all resource settings. High coherence.
**Role of hospital in acceptability of interventions to reduce unnecessary CS:** Type of hospital (public, private, university teaching, regional referral) and degree of autonomy over management were reported as important determinants of actual CS rates in organisation or facilities. The importance of relationships between hospitals and out-of-hospital care providers to facilitate referral in if needed was also noted.	36,42–44, 46,47,55	Moderate confidence	7 studies with no to moderate methodological limitations. Thick data from MICs. One LIC study. Moderate coherence.
**Apathy to change rooted in the interdependency of overall structure and complexity of healthcare systems:** Across the world, in HIC, MIC and LICs stakeholders’ reported resistance to change rooted in the belief that the reasons for caesarean section rates are a hugely complicated series of events, including both clinical and non-clinical factors.	28,43,46, 47,52–55,57–60	Low confidence	10 studies with minor to moderate methodological limitations. Only thin data from across 4 geographical regions with only moderate coherence.
**Summary theme 2: Human and cultural factors**			
**Strength of multi-disciplinary collaboration, teamwork, communication, role demarcation and respect across maternity care system:** Policy makers and practising health professionals, across HIC and MICs reported effective teamwork as a key component to tackling unnecessary CS. Across setting organisations with the highest CS rates reported experiencing more challenges in achieving multi-disciplinary working within and between midwives and obstetricians, in organisational culture and in policy documents.	40,42,46,47,49,50–58,60–62	High confidence	15 studies, most with minor methodological limitations. Some very thick data from HICs and MICs. Data from all resource settings and 5 geographical regions. High coherence.
**Attitudes towards risks, benefits and organisational rates of CS:** In HIC and MICs health professionals had varying attitudes towards the value of CS. Some claimed a lack of awareness of any ill-effects of CS or their facility’s CS rate, others acknowledged their rates where high and risks existed but considered them “ignorable”, while some expressed specific concerns about anaesthetic risks, surgical complications, increased recovery time, cost longer term consequences for women. Women in Ghana were aware both that access to a health insurance scheme that gave them free maternity care could benefit them if they needed a CS, but also that this lead to an increase in CS rates and increased morbidity for some women.	36,39,42,46,47,50–56,59,61	High confidence	12 studies with minor methodological limitations. Some thick data from across 5 geographical regions. High coherence.
**Belief quality of care for women is compromised or enhanced by reducing unnecessary CS:** In HIC and MICs inertia to change amongst some health professionals was rooted in perceptions of women’s preferences for obstetric-led care and CS. Some health professionals also perceived women as lacking in antenatal preparation for labour and vaginal birth. In the UK, US and Canada in organisations where care was actively focused on the promotion of normal birth health professionals reported positive impacts on women’s experience.	27,39–42,46–55,57–62	High confidence	19 studies with minor methodological limitations. Thick data from 5 geographical regions. High coherence with variations in data explained by degree of concern. Studies predominantly from MICs and HICs with high CS rates.
**Valuing of human-to-human care during childbirth (including emotional labour, companionship and advocate for woman):** In HICs and one MIC women reported welcoming labour support from doulas or midwives. Health professionals talked about the importance of partner support and one-to-one midwifery/nursing care in HICs where these were available to many women. In MIC settings the value of labour support was recognised but availability was limited by too few midwives and inadequate facilities for partners to accompany women during labour.	36,39,40,41,46,47, 49,52–57, 61,62	Moderate confidence	13 studies with no to moderate methodological limitations. Thick data from 4 geographical regions. Studies only from MICs and HICs. No LICs. Uncertain confidence in LICs. Moderate coherence.
**Concerns about culture of intervention in childbirth:** In HICs and MICs some stakeholders reported how the medicalization of childbirth can devalue it as a physiological process. Where interventionist organisational cultures were acknowledged as a problem, midwives and obstetricians talked about how it limited both their opportunities to fulfil their role optimally, and the opportunities for women to experience normal pregnancy and childbirth.	36,39,42,46,47,49, 50,52–57, 61,62	Moderate confidence	13 studies with no or minor methodological limitations. Thick data from 4 geographical regions. High coherence. Studies only from MICs and HICs. No LICs. Uncertain confidence in LICs.
**Shifts to standardise care were widely desired but not universally acceptable in practice:** Across HICs and MICs many health professionals reported a desire for more standardised tools in the form of guidelines, care pathways, screening tools and audit. There were discrepancies between what policy makers said existed and clinicians said they were aware of. Where interventions were implemented they were variously received as legitimising existing good practice and supportive of clinical judgement; empowering for midwives faced with pressure from obstetricians against a shift from medical to midwifery-led care; or actively resisted, their formulation challenged (in terms of their evidence-base, or tick-box approach) and experienced as constraining of clinical judgement. The burden of tools (IT and other) to audit and record standardised processes, and the time this took away from direct hands on care, was also noted.	40,42,50–55,57,58	Moderate confidence	8 studies with minor to significant methodological limitations. Very thin data from one study in LICs. High coherence.
**Attitudes towards in-practice use of best-evidence:** In HICs attitudes towards evidence varied. In some organisational cultures evidence was embraced as part of the drive for continuous quality improvement, whereas in others the quality of evidence underpinning programmes was questioned and/or organisations were selective in their use, particularly of evidence for midwifery-led care models. In MICs the desire for practice to be evidence-based was commonly discussed but felt to be not achievable in practice because of system limitations (resource, culture of intervention).	40,42,47,50, 52–54,55	Low confidence	6 studies, most with no or minor methodological limitations. Data thin and only from HICs and MICs. Moderate coherence.
**Summary theme 3: Mechanisms of effect for change factors**			
**Effective leadership, stakeholder involvement and ownership:** Stakeholders reported the need for interventions to be publically given high priority across organisations, facilities and systems (including positive media coverage) with respected, identifiable leaders at every level (both top-down and within and across professional peer-groups) to make cultural change happen. All participants with a stake in maternity care (women, obstetricians, family doctors, midwives, policy makers, managers) reported the need for involvement in the development and implementation of interventions with opposition often stemming from feelings of exclusion, alienation and lack of ownership. Key considerations here were the degree of resistance encountered (see also local context) without effective, sustainable leadership, overt organisational buy-in, no mandatory requirement to change or long-term accountability for CS rates. Hospitals that achieved success in reducing rates identified nursing and medical leaders who endorsed and championed the project, made change an institution wide policy priority, not pilot or developmental. In a few MICs the need for a National Task Force with obstetric and midwifery representation was noted (Iran, Lebanon, Chile).	28,40,42,47, 50–58,60–62	High confidence	14 studies with no to moderate methodological limitations. Thick data from 4 geographical regions and across resource settings. High coherence.
**Health professionals’ attitudes towards changing workloads:** Across the world, in all resource settings implementing interventions had consequences for everyday workloads. Insufficient resources for designated staff or dedicated time to work towards the successful implementation of interventions was viewed negatively the world over. In the UK MLC initiatives that made midwives the lead professional increased individual midwives workload (rather than putting more midwives in the system) and changed the nature of doctor’s workload by limiting their interpersonal involvement with women and making it harder for them to anticipate demand. In MICs increasing workloads of midwives to the point where they were stretched was reported to be a factor increasing CS rates, not reducing them as midwives came under intense pressure to free up beds.	42,46,47,49, 50–58,60,61	High confidence	13 studies, most with no or minor methodological limitations. Thick data from across geographical regions and resource settings. High coherence.
**Fears about safely of reducing CS rates and skills and confidence to deliver normal birth amongst obstetricians, midwives and women:** In HICs and MICs some obstetricians and some midwives raised concerns about their professions competency to change and deliver more women vaginally, while in HIC settings with lower CS rates midwives and obstetricians were more confident that normal birth is where midwifery’s strength lies and obstetric colleagues were well-trained to deal with complications should they arise (i.e. high level surgical/operative skills, vaginal breech skills, and forceps skills). In MICs decision-makers cited several advantages to vaginal birth, while physicians focused on the disadvantages favouring CS to prevent any complications arising, particularly amongst women who live in isolated areas with little access to specialists should they need one. A lack of confidence in normal birth on the part of women was also noted.	27,36,39,40,42,46, 47,49, 50,52–55, 57,61, 62	Moderate confidence	14 studies with no or minor methodological limitations. Thick data from HICs and MICs. No data from LIC resource settings. High coherence.
**Education and training that prioritises normal birth and continuous quality improvement:** Various education needs in order to implement system change and reduce unnecessary caesarean section were identified by stakeholders. These included better prenatal education for women and better training of health professionals in clinical skills, clinical audit and the programme content of a specific interventions targeted to reduce unnecessary CS.	40,42,46, 47, 55,56, 57,61	Low confidence	8 studies with minor to moderate methodological limitations. Thin data from 4 geographical regions. No LICs. Uncertain coherence.
**Importance of understanding local context, culture and existing initiatives that influence how favourable an organisation, facility or system is to reducing unnecessary CS:** Stakeholders views (policy makers, healthcare managers, health professionals and women) highlighted the importance of understanding local context in negotiating support and resistance to change. Understanding current practice patterns (including maternal request for CS), pre-existing initiatives (financial strategies and incentives, other guidelines, evidence-based practice, local audit priorities), and the importance of stakeholder involvement in the design of interventions were discussed with understanding where an organisation, facility or system is currently at as fundamental to the acceptability of an intervention.	27,40,42, 47–58,60–62	High confidence	16 studies with minor methodological limitations. Thick data from 6 geographical regions, 12 countries and all resource settings. High coherence.
**Adaptive, multi-faceted interventions with local ‘tinkering’ acknowledged as components in success (or failure):** Stakeholders views and experiences of interventions show how they are not implemented in isolation. They are continuously and creatively negotiated on-the-ground in ways not easily captured or anticipated (administrator pride in revenue from increased CSs, length of time to bring about change different in different contexts). The factors that contributed to an interventions effectiveness were often opportunistic (i.e. capitalised on other developments in other areas of the health system) and reflected a change in culture, rather than adherence to a particular checklist or rigid protocol. They also had to have built-in mechanisms for multi-disciplinary collaboration and communication for continuous quality improvement that were adaptive to local ‘tinkering’ (i.e. women previously identified as "normal" classified as potentially "at risk", meaning the increased status of midwifery work was compromised by a reduced scope of practice in programmes for MLC or normal birth in HIC and MICs).	28,39,40, 42,46,47, 50–54, 56,58–61	Moderate confidence	14 studies with moderate to minor methodological limitations. Thin data from 5 geographical regions and all resource settings. Moderate coherence.

Nineteen SoF statements were derived. They mapped onto three distinct themes (Table 3): Health system, organizational and structural factors (6 SoFs); Human and cultural factors (7 SoFs); and Mechanisms of effect to achieve change factors (6 SoFs).

**Table 3:** Initial concepts, emergent themes, final themes and supporting quotes.

Initial concepts	Emergent themes	Papers	Illustrative quotes	Final summary themes
Power of medical profession	Balance of power between stakeholders: Professional power, roles and relationships	42,46,47, 49,50,52–54,57,58, 60,61,62	“It is very difficult to work in this structure where doctors always have the first place.” (Binfa 2013:1155) “There were lots of people who had lots of doubts about it [normal labour pathway] for lots of reasons—whether this was appropriate for midwives?” (Hunter 2010a:229) “I just feel that we’ve [midwives and obstetricians have] got different agendas.” (Midwife, Cheyne 2015:336) “What I have witnessed in medical assemblies during these years was that we were the last; our efforts are not rewarded neither from financially or spiritually. And not recognising our profession and its hardships, takes all the encouragement away.” (Midwife, Janani 2015:1376, Iran). “The law does not protect midwives. Physicians are more protected by law.” (Midwife, Yazdizadeh 2011:6) The (fee reduction) policy was well-adopted by the hospital managers. Nurses and midwives in general perceived the policy as a positive one… doctors, and especially specialists were often found to use their power position to implement the policy half-heartedly or to change it to their advantage. (Witter 2016:12) “… to have the hierarchy of the doctors and nurses be less pronounced.” (Mother, Kennedy 2016:342)	Health system, organizational and structural factors
Power of midwifery profession				
Relationships with women				
Facilitator of access to CS for women and midwives	Fee exemption/reduction policies as mediators of access to necessary and unnecessary CS	28,43,44, 58,59,60	"There are more referrals thanks to the exemptions policy. Matrones no longer keep back in the cases women who lack the means‴ (Facility Key Informant, Witter 2008) You demand total non-charging, but it doesn’t happen like that at all. It’s not the state that is in charge of the health centres.’ (man, Witter 2009:6) “It has created too much robbery.” (Husband, Lange 2016:57) “Sometimes after the C-section, the sore can become infected…even when the sore heals… It reduces the strength and economic activities that you can do (” (Mother, Rishworth 2016:e123) L’argent des césariennes: une bouffée d’oxygène pour les hôpitaux: ('cesarean income; a breath of oxygen for the hospitals') (Mbaye 2011:216)	
Short and long term costs of free for families				
CS revenue as a means of income generation for facilities				
Health insurance, women’s choice and/or clinicians’ indication	Health insurance reform as a mediator of access to necessary and unnecessary CS	27,29,47, 48	“The charge for CS was high. Under profit driving, CS rate increased.” (Zhu, 2013) “Proportional reimbursement may have some effect on the caesarean delivery rate. Caesarean delivery would cost 200 yuan and women could get nearly 1000 yuan back.” (Health Manager, Huang 2012:7) “In Iran, the insurance companies sign a contract with healthcare providers and pay them rather than compensating the service itself. Considering the fact that the service provided by the midwives is not covered by insurance companies, expectant moms prefer to go to a specialist. In this situation the rate of additional interventions and C-sections would increase.” (Yazdizadeh 2011:4)	
Power of insurance companies				
Built environment as barrier or facilitator to a positive labour and birth experience	Birth environment, efficiency concerns and organisational logistics	28,36,39, 40–42,46, 47,49, 51,55–58,61,62	Worked to improve birth environment–but beds got moved back. (Marshall 2015:336) They [the midwives] view the home setting and the presence of valued and welcomed friends and relatives as key elements… (Sakala 1993:1242) “…one labour room was shared between three mothers. One of them had given birth 30 minutes ago and the baby was in the Kangaroo position on the mother's chest, one of them was expected to be in full dilation, and one of them was in the early stages of labour". (Field note, Behruzi 2010:11) “Contrary to international standards, the size of our labor rooms have reduced and they have been converted into operating rooms over time. . . .” (Midwife) …“These facilities are old fashioned and designed for group labor rooms, and therefore should be modified.” (Physician) (Yazdizadeh 2011:9) “The Labor room lacked appropriate air-conditioning and adequate lighting… equipment and facilities for the use of non-pharmacological methods of pain relief were not enough.” (Janani 2015:1376)	
Time and resource constraints on labour progress				
Organisational policy priorities and use of room(s)				
Type of hospital (independent/ private or public)	Role of hospital in acceptability of interventions to reduce unnecessary CS	36,42–44, 46,47,55	““…independent hospitals do anything to have higher incomes;” (Yazdizadeh 2011:7) “In the private sector, providers are reimbursed approximately $700 for normal childbirth and $1,500 for caesarean section.” (Colomar 2014:2388) “This hospital accepts trainees, and we cannot stay with mothers all the time.” [Behruzi 2010:12] “The big women’s and children’s hospitals are teaching hospitals, and are training sites for residents and specialists [who need surgical experience], and that is obviously going to increase the caesarean rate.” (Colomar 2014:2385) The absence of full-time specialists in teaching hospitals and the fact that 1st and 2nd year residents are responsible for the delivery…have contributed to an increase in the C-section rate in these hospitals.” (Yazdizadeh 2011:8) ‘Women living in urban areas benefit most from the policy as everything is centralized in the districts.” (Witter 2009:8)	
Designation of hospital/facility (regional, teaching, district, rural)				
Complexity of system (people, policies, place) as barrier to change	Apathy to change rooted in the interdependency of overall structure and complexity of healthcare system	28,43,46, 47,52–55,57–60	“It is not one thing, it’s the overall structure, which includes midwives, doctors, junior staff …” (Doctor, Hunter 2014:731) “Since the policy came into force we have not received a single cent in reimbursement. In any case, we do not really know what procedure to follow for reimbursement." (Witter 2008:98) “Patients do not receive the required care during pregnancy and therefore the high-risk cases are not detected;” “Whenever you try to modify the system you face a problem.” (Yazdizadeh 2011:9)	
Complexity of clinical and non-clinical factors converging				
Cross-disciplinary shared purpose and commitment to normal birth and/or CS rate reduction	Strength of multi-disciplinary collaboration, teamwork, communication, role demarcation and respect across maternity care system	40,42,46, 47,49,50–58,60–62	“I do think we’ve made good progress with it [multidisciplinary working].” (Marshall 2015:337) "This hospital provides more natural births. Many women choose this hospital for natural births… We believe that only some women need epidurals, for example, anxious women …". (Paediatrician, Behruzi 2010:11) “In this practice I have appropriate professional autonomy and respect… so I trust that my consultants are available and… otherwise in a normal situation appropriately disinterested.” (Midwife, Kennedy 2016:342). “The midwife can have the main role in the labor process unless the patient asks to have a physician at her bedside… In these situations, the physician only interferes if a problem occurs. The specialist can also ask a midwife to stay at the bedside of her own patient until it’s delivery time and thereafter the physician can carry out the process herself.” (Yazdizadeh 2011:8) Team working had suffered as a result [of implementation of normal labour pathway]; as a midwife commented: ‘‘It makes it ‘us and them”‘ (Hunter 2010b:233)	Human and cultural factors
Respectful team working				
Antagonistic team working				
CS as cultural norm	Attitudes towards risks, benefits and rates of CS	36,39,42, 46,47,50–56,59,61	Perception that CS is normal. (Kennedy 2016:340) “C-section is becoming more common and stylish these days” (p.11); “C-section for multiparous women is associated with limitations and various complications but if the mother intends to have a single or at the most two deliveries not many complications arise;” “Despite the reduced number of pregnancies, women undergo surgeries due to various other reasons in which the adhesions caused by previous C-sections might become troublesome.” (Yazdizadeh 2011:6) “Too many Caesareans is not nice.” (Doctor, Hunter 2014:731) “C-Sections are becoming too much.” (Woman, Rishworth, 2016:e122) “The truth is that we do not have statistics regarding caesarean complications, which could show a fatal outcome or anything like that.” (Physician, Colomar, 2014:2385)	
CS rate and outcomes as cause for concern				
Lack of knowledge about CS rates and outcomes				
Women as key stakeholders to system change	Belief quality of care for women is compromised or enhanced by reducing unnecessary CS	27,39–42,46–55,57–62	There was no public consultation with maternity service users (client involvement depended solely on the service user group representative on the steering group) (Hunter 2010a:231) ‘‘It is requested a lot (cesarean)…” (Colomar 2014:2385) “Many women demand Caesarean section during admission even before entering the labor room…” (Janani 2015: 1377) “…we should assure mothers that C-section would be performed if needed, adding that vaginal delivery would not be our choice if its risks outweigh its benefits. In other words, we choose the method which is best for both the mother and baby.” (Yadizadeh 2011:11) “Belief that labour is a normal event.” (OWHC 2000:45)	
Belief women want CS and/or it offers a more positive birth experience				
Belief in labour and birth as normal				
Attitudes towards 1:1 labour care	Value of human-to-human care during childbirth (including emotional labour, companionship and advocate for woman)	36,39,40, 41,46,47, 49,52–57,61,62	“Commitment to 1:1 labour support.” (44) “Philosophy of a natural experience; being a support person/ advocate rather than technician.” (OWHC 2000:45) “The companion talks with the patient and this reduces the patient’s stress. They go to the next step together gradually. But considering the fact that we don’t have enough human resources in the field, the quality of communication between the midwife and the mother has declined.” (Yazdizadeh 2011:8) ‘‘It is a facilitating factor that the companions are already immersed in the process of prenatal care and, therefore, care in labor…” (Colomar 2014:2388) “I was confused before she came to me. I was having a lot of pain, but when she came to me I was active and happy.” (Shelp 41:7) “Alone, I wouldn’t have known what to do.” (mother with doula) “I would have liked my mother or my husband to be there, to have some support, to feel someone’s affection, to feel I was important to someone.” (Mother without doula, Campero 1998:401).	
Value of companion/support person				
Belief too much unnecessary intervention in childbirth/concern cultural norm	Concerns about culture of intervention in childbirth	36,39,42, 46,47,49, 50,52–57,61,62	“An expectant mother who is being monitored… receiving IV-solutions…, catheterized… These unnecessary interventions increase the risk of C-section.” (Yazdizadeh 2011:8,Iran) Humanized birth is not a case without any medical intervention. Sometimes we need medication [. . .] we should marry humanized birth with medical intervention just by explanation, communication and the maintaining confidence". (Midwife, Behruzi 2010:9, Japan) “They’ve [doctors] got to be seen to be doing things. They get their hand in, rather than say ‘Hang on a minute, just step back. Let her be given a bit longer.’ (Manager, Hunter 2014, UK)	
Intervention when necessary				
Desirability of guidelines and clinical governance (audit)	Shifts to standardise care were widely desired but not universally acceptable in practice	40,42,50–55,57,58	“We are very clear on that… in Latin America and Central America the incidence [of caesarean births] decreased when a good protocol was established… “‘Despite being the directors of health we do not have much control over the private sector, and we have problems; even in overseeing our own units, we make a great effort but we have very few staff to monitor the private units” (Colomar 2014:2388) The majority of participants believed that some of the protocols relayed to the hospitals did not contain enough integrity and functionality and flaws in their implementation can cause problems. (Janani 2015:1376) “It’s a bit too dictatorial for me … You don’t need instructions telling you how labour progresses. Things like that should be part of your midwifery practice.” During observational fieldwork, no midwives were seen consulting the pathway as a decision-making guide. Use of clinical judgment was evident. (Hunter 2010b:232	
Acceptability in practice				
Embracing of evidence	Attitudes towards in-practice use of best-evidence	40,42,47, 50,52–54,55	“Embracing of evidence and the drive to continually improve.” (OWHC 2000:45) “It does give you a little bit of ammunition.” It’s written down and because it’s coming from research, you’ve got all the references in front of you as to what type of research has been used and it sort of … just backs you up;” “We’re swapping one lot of vague-ish evidence for another lot of vague-ish evidence–and wait and see if anything goes wrong or not.” (Hunter 2014:728–9) “Evidence-based medicine, which we are trying to follow in our practice, stresses that one of the vaginal delivery complications is the relaxation[of the vagina], but do we inform our patients about the complications associated with C-section as well? Never. Do we inform mothers about possible side effects of the anaesthetic agents, injuries sustained to the genitourinary system, more bleeding, higher infection rates and more infant-related problems associated with C-section? (Yazdizadeh 2011:10)	
Scepticism of evidence				
Selective use of evidence				
Leadership	Effective leadership, stakeholder involvement and ownership	28,40,42, 47,50–58,60–62	“Commitment of the management team to true quality of care, i.e. the patient comes first.” “Support from management to deal with change, stress and conflict management;” “Institutional support for the program;” “Strong leadership role model within a shared governance model.” (OWHC 2000:45) ‘Hospitals that achieved success in reducing their rates identified nursing and medical leaders who endorsed and championed the project.” (Dunn 2013:310) “…the staff are briefed for ten minutes a day on what's on the board, so therefore everybody hopefully is buying in to providing better care, knowing our results and what we should be pursuing to make our results even better. There's also a section on the board which is called Bright Ideas, and staff are expected to contribute to a bright idea.” (Head of Midwifery: Marshall 2015:335) ““One of the problems we have is that by presenting a program, we cannot expect the program to be implemented in the best way. The managers should perceive the weaknesses and strengths of the program, personnel’s function, punish offenders, and reward good workers, which should not be necessarily financial. We become disappointed when we do not have these.” (Janani 2015:1376) “We kind of sit there waiting for the next step or for them to tell us what’s going on; and I think if we could change that culture.” (Mother, Kennedy 2016:341)	Mechanisms of effect for change factors
Buy in within and across professions, organisations and systems				
Feelings of alienation, exclusion and exhaustion				
Listening to mothers				
Attitudes towards redefining professional role boundaries	Attitudes towards changing workloads, time and resource	42,46,47, 49,50–58,60,61	“There is a loss of that relationship [with women] and also the loss of being present with more normal deliveries… (Hunter 2014:733) ‘‘I don’t know if anybody. had any idea what it would involve or what a big project it was or how much time it would take you…” (p.230)… The audit had been “tagged on the end” No additional resources or budget were available (p.231) To be effective, time must be allocated for these [steering Group membership] roles rather than adding to existing workloads. (Hunter 2010a:232) Factors facilitating this [the success of the Toolkit] included: recognising the need for staff dedicated to the project with protected time and resources (Marshall 2015:338) “We have a lot of work to do and just don't have time [for humanised care during labour].” (Behruzi 2010:13) “…our center is too crowded and this is an important factor. We send expectant mothers who can be C-sectioned rapidly to the operation room in order to have more vacant beds.” (Yazdizadeh 2011:7) “The number of midwifes in each shift in proportion to the number of patients is really low…” (Janani 2015: 1376) “The physician goes to the hospital in the morning and to the clinic in the afternoon… I can’t revisit my patient in the hospital at 10pm to carry out a vaginal delivery.” (Yazdizadeh 2011:10)	
Additional work involved as direct consequence of intervention				
Pressures on everyday workloads				
Fear, unpredictability and safety of vaginal birth	Fears about safely of reducing CS rates and skills and confidence to deliver normal birth amongst obstetricians, midwives and women	27,36,39, 40,42,46, 47,49, 50,52–55,57,61, 62	“We have to do [caesarean section] because pregnant women and their family think caesarean section can guarantee safety of both mother and baby.” (Liu 2010) “From what I understand, a normal care pathway means that this patient is presumed absolutely normal and will have absolutely normal labour, which I have a big reservation about because in labour, even if the patient had no problems before, you never know until the patient is delivered and the placenta is out… you see the problem with obstetrics is that some of them are very, very dicey and dangerous…” (Doctor) “…women will get on and do it themselves if you give them a chance to do it. “(Hunter 2014:732) “I know how to get [babies] out,” and “women are built to open up there” (Sakala 1993:1240)	
Skills and confidence in normal birth				
Training, education and experience of normal birth	Education and training that prioritises normal birth and continuous quality improvement	40,42,46, 47,55,56, 57,61	“‘ …their [obstetricians] view was that perhaps midwives weren’t using their professional judgement correctly, that they were leaving ladies too long without intervening, whereas our view was that maybe sometimes they were intervening too soon …” (Head of Midwifery) “I think that people are reluctant to change….Some of the consultants are very medicalised, and some of the midwives for that matter, quite tough to get on to side… Not everybody needs to be on CTGs… (Clinical midwife) (Marshall 2015:327) “In the past few years many obstetricians have never had the opportunity to do a vaginal delivery. The knowledge of a first year resident regarding the procedure is similar to that of an intern. Residents learn the process of natural delivery during the first year but by the time they have learned how to deal with physiologic labor, the year ends and a new unskilled group becomes responsible for the whole thing.” (Yazdizadeh 2011) “Education sessions were presented by paediatricians or obstetricians to communicate site-specific rates to the team, to discuss the evidence and the risks to neonates [of elective repeat CS before 39 weeks, and to garner buy-in for changes across the organisation. (Dunn 2013:311) “A commitment to continuous quality improvement such that great effort has been made to ensure that staff are aware of national standards and guidelines, and are encouraged to work collaboratively to decide how to get there.” (OWHC 2000:45)	
Continued professional development and organisational commitment to continuous quality improvement				
Extent practices already in place	Importance of understanding local context, culture and existing initiatives that influence how favourable an organisation, facility or system is to reducing unnecessary CS	27,40,42, 47–58,60–62	Most practices in relation to KCND were already in place. (Site B) [in contrast to a] Highly ‘medicalised’ model of care (Site C) (Cheyne 2013:1115) “We have always been interested in providing humanistic care, even before this guide was implemented.” (Midwife) “… to me this is the same assistance I received during my last delivery, nothing has changed.” (woman) (Binfa 2013:1153) These strategies were not effective… The model was initiated without acknowledging the socio-cultural characteristics of each regional context and ignoring local realities regarding the attitudes of each regional health team. (Binfa 2016:60) “For us to change… at first it was hard, but… we have begun to accept, we try…” (Colomar 2014:2388)	
Professional opposition				
Concurrent guidelines, policies and strategies				
Opportunistic implementation factors	Adaptive, multi-faceted interventions with local ‘tinkering’ acknowledged as components in success (or failure)	28,39,40, 42,46,47, 50–54, 56,58–61	The idea for developing the clinical pathway appears to have been largely opportunistic. (Hunter 2010:228) The decision to expand the policy to the regional hospitals in the remaining regions was, according to one Kl, informed by budget under-spend (Witter 2008:97) Physicians document an "accepted" reason making accurate assessment of underlying reason rather impossible (Yazdizadeh 2011) Participation as a trial site provided opportunity to do things differently (Camperio 1998)	
Local creativity and adaptation				

### Summary theme 1. Power, place and perverse incentives: Health system, organizational and structural factors

This theme encapsulates how structural health system, facility management and organizational factors that exist at an aggregate-level impact the values of stakeholders, and shape individual views of the feasibility, or otherwise, of interventions to reduce unnecessary caesarean section.

#### Supporting and challenging professional power, roles and relationships (SoFs1)

The power of the medical profession was perceived as an important barrier to overcome where doctors believed their professional identity and the safety of women was compromised by relinquishing lead professional responsibility to midwives. [50,52–54] Some midwives expressed similar concerns where midwifery confidence, skills and support were low within specific organisations [46,50,52–54] and systems. [46,47,49,52–54] As explained by this midwife in Chile, “*Neither midwives nor women are empowered enough to question a medical prescription*.*” (*[49]: p.1153). Women too reported observing the negative effects of power differentials between doctors and midwives. [61] In 11 studies, reported in 13 papers [42,46,47,49,50,52–54,57,58,60–62] interventions, including initiatives to promote physiological birth in Iran [57], hospital primary vaginal birth in the US [61], normal labour and birth in the UK [50,52–54] and the humanization of birth in Japan [46] and Chile, [49,62] challenged the structural balance of power between stakeholders. In UK organisations where a more equal balance of power did exist between women, midwives, family doctors, and obstetrician, there was some evidence that midwifery-led staffing interventions to keep birth normal and reduce caesarean section empowered midwives to work more autonomously [50,52–54] by *“…sort of put[ting] a little tag on that woman as a way of saying ‘leave her alone’*, *which I think some doctors respect*, *and some don’t*” ([53]:p.232). A further perspective on power, roles and relationships between stakeholders was offered by a nurse in the US who said; *“My job is to empower them [women]*. *I don’t need to feel powerful…” (*[61]:p.341).

#### Perverse incentives, fee exemption, fee reduction and health insurance reform (SoFs 2, 3)

Financial incentives, for hospitals, doctors, or women, either to reduce caesareans, or to increase access to caesarean section when needed, were not always perceived to have had the desired effect. In one study from China insurance reform was not believed to be as influential on caesarean section rates as women’s preferences for caesareans. [48] From low- and middle-income countries there was evidence that financing structures, in the form of fee exemption policies [28,43,44,58–60] and insurance reform, [27,29,47,48] were mediators of access to both necessary caesarean section and unnecessary caesarean section. Whether financial interventions were successful or not was mediated by local philosophies of maternity care; inter-professional and inter-personal relationships; staff motivation to work with women or with the organisation, or simply for an income; and the expectations and demands of local women, families and communities.

For example, in a study from Senegal, the intervention was government payments for each caesarean performed, with the intention of ensuring that necessary caesarean section was accessible to all. All participants in the study (including women, medical and midwifery staff) perceived all caesareanss conducted as necessary. In a highly telling interview, an administrator spoke of the increased revenue generated by this policy as the *cash-cow* for the hospital; the “*vaches laitières des hôpitaux*.*” (*[28]:p216) It was seen as a source of pride for the obstetric department, providing them with power and influence in the hospital as a whole. In Iran, insurance policy change was met with scepticism by health professionals, amid concerns that women who need a caesarean section may no longer get one, or that there might be a paradoxical increase in the misreporting of indications for caesarean section to satisfy amended insurance criteria. [47]

#### Birth environment, efficiency concerns, and organisational logistics (SoFs 4)

In 16 studies, stakeholders talked about the built environment (i.e. physical space, facilities), efficiency (i.e. time constraints on labour and staff) and/or logistical concerns (i.e. availability of equipment, theatre access) as powerful mediators of barriers or facilitators to reducing unnecessary caesarean section. [28,36,39,40–42,46,47,49,51,55–58,61,62] In high-income countries where *“quick win”* changes had been made to labour and delivery rooms to encourage normal labour and birth, the priority an organisation gave to maintaining them was fundamental to their effectiveness in reducing caesarean section rates. [56,61] This included changes to in-room facilities for labour and guaranteed access to operating theatres when necessary. [51,56,61] One study reported midwives’ views about how birth in a home setting reduces unnecessary caesarean section, [36], citing the absence of restrictions on women’s movements, environmental comforts, and efficiency concerns evident in the other 15 studies of institutional birth contributing to this SoFs. In middle-income countries inadequate facilities (lighting, bathrooms, air-conditioning and shared delivery areas), or the actual conversion of delivery rooms into operating theatres, were reported as important barriers. [42,47,55,57] The need to consider the birth environment as comprising of material facilities, but also material relations between humans and systems was evident within and between studies, and across resource settings.

#### Role of hospital: philosophies, purpose and structures (SoFs 5)

Type of hospital, such as whether the hospital was in the public or private sector of care, a university teaching hospital and/or a regional referral centre, was perceived by stakeholders to influence the acceptability and feasibility of specific interventions to reduce caesarean section rates. [27,36,42–44,46,47,55] This could simply be a consequence of different financing structures, clinical policies, and the working environment. However, it could also be due to the power of the predominant philosophy of pregnancy and childbirth, based on perceptions of the purpose of the particular kind of unit. For example, being a University affiliated hospital was viewed by some stakeholders as a potential barrier to caesarean section rate reduction because of the lack of continuity of care and interpersonal relationships due to task and intervention orientated pressures, [46], or to the organisational need for medical residents to take responsibility for births, in preference to midwives [47]. In contrast, where larger or more academic hospitals were associated with better governance structures, this was perceived to be associated with low caesarean section rates, as in the case of Lebanon, where it was reported that caesarean section were low because *rigorous audit systems* [are] *more common in teaching hospitals*. ([42]:p.45)

#### Apathy to change, interdependency and complexity of system (SoFs 6)

Across settings, the complexity of the healthcare system, were clinical and non-clinical factors inevitably converge was perceived as a barrier to simple, standardised interventions to reduce unnecessary caesarean section. [28,43,46,47,52–55,57–60]^.^ This was partly due to the powerful impact of non-clinical factors, such as management processes, rules, regulations, and conflicting strategies. [46,47] For example, the interdependency of the British National Health Service’s internal structures and workforce (midwives, obstetricians, junior doctors), and *“the hugely complex series of events”*, contributing to high rates of intervention in pregnancy and childbirth, meant many participants reported that achieving higher rates of normal birth and lower rates of caesarean section was *unlikely to be effectively addressed by the apparently simple solution of a clinical pathway*.*(*[53]:p231) In Nicaragua, healthcare providers spoke of high CS rates as a way of compensating for the multi-dimensional weaknesses in their health system (including insufficient human resource, material resource, or coverage). [55] This was evident in other middle- and low-income countries where antenatal care was absent, communication between all levels of the system, and between the system, staff, and women, was deficient, and infrastructural and geographic challenges of reaching skilled labour care existed. [43,47,58,59]

### Summary theme 2: Norms and human relationships: Human and cultural factors

This theme captures the way in which the culture in and of organisations, facilities and systems may impact stakeholder views of interventions to reduce unnecessary caesarean section. This included the forms of behaviours that are learnt across generations, and those that are characteristic of a particular time and place.

#### Multi-disciplinary collaboration, role demarcation and respect (SoFs 7)

In 15 studies, the strength of multi-disciplinary teamwork in an organisation or system was reported to be an important barrier to or facilitator of caesarean section rate reduction. [40,42,46,47,49–58,60–62]. The kind of teamwork that mattered was less about working directly on the caesarean section rate, and more about the general ethos and atmosphere of mutual respect. Stakeholders from organisations or systems with high caesarean section rates said working relationships between professionals were poor, with collaboration, communication, and respectful role demarcation between professionals lacking. [56,57,61] As expressed by this Iranian midwife *“in many cases of care, we need to ask other colleagues to do the examination, or other things to help but unfortunately, some colleagues do not believe in helping their colleagues” ([57]:p.1277)* ^.^ In contrast, stakeholders working within organisations with low caesarean section rates valued “*working together as a team*, *knowing that everyone’s voice will be heard*, *and action is taken at every level of the organization*.*” (*[40]:p.45) One explanation as to why respectful teamwork may contribute to lower organisational caesarean section rates was offered by a UK midwifery manager: *“everybody has greater awareness; consultants*, *registrars*, *SHOs*, *ultrasonographers*, *student midwives*, *student nurses*, *anaesthetists… they all bring a different perspective and they also take credibility back to their own peer group*.*”* ([56]:p.337)

#### Whose risks, whose benefits’? Attitudes towards risks, benefits and rates of caesarean section (SoFs 8)

Important differences in stakeholder attitudes towards caesarean section were reported. [36,39,42,46,47,50–56,59,61] Within and between studies, some health professionals described a lack of knowledge about caesarean section rates, indications or outcomes [42,51,55] while other health professionals and women perceived caesarean section as *“normal”*. [61] Some health professionals acknowledged caesarean section rates were (too) high locally, and that this might increase risks, but perceived them to be less, or no more severe, than the risks associated with vaginal delivery for mother or infant. [42,47,51,55]. In one study some specialists *claimed the complications secondary to C-section are ignorable (*[47]:p.6), while other health professionals reported concerns about anaesthetic risks, surgical complications, increased recovery time, cost, and longer term consequences for women. [46,47] In a US study, an obstetrician summed up how attitudes towards caesarean section are shaped by cultural context, at the same time as suggesting the potential of human agency; “*People are starting to think; are we really doing the right thing*? *And I think the answer is clearly no* … *I can’t believe that evolution is pushing us into the operating room*. *I think* *we’re* *pushing ourselves into the operating room… it’s almost like the perfect storm. You’re going to pay me more, I get to worry less, you’re not going to sue me, and I’ll be done in an hour.” ([61]:p.342)* Women also had varied views about birth method, some of which were resonant with those of health professionals. One important difference in women’s views was the embodiment of living with the health consequences of caesarean section. For example, in the context of Ghana’s subsistence culture, one woman said *“the C-section itself becomes a disease*.*”* ([59]:p.e123)

#### Beliefs about quality of care mediated by beliefs about caesarean section (SoFs 9)

Related to stakeholders’ attitudes concerning caesarean section, were their varying beliefs about whether care quality is compromised or enhanced by reducing caesareans. [27,39–42,46–55,57–62] In the UK, US and Canada in organisations where care was focused on the promotion of normal birth and reducing, or maintaining, low caesarean section rates, some health professionals viewed this as having a positive impact on women’s birth experiences and quality of care. [40,59,61] However, within these studies [59,61] where a specific facility’s organisational culture endorsed maternal request caesarean section, and across other studies from high- and middle-income countries, health professionals’ inertia to change was based on the belief that women increasingly want caesareans and are inadequately prepared for labour and vaginal birth. [27,42,46–48,51,55,57,59,61]. Twelve studies reported women’s views, [27,39,41,42,46,48,49,58–62] including their choice of caesarean section and lack of antenatal education about labour, vaginal birth and caesareans. Two studies noted that maternity service users’ views about the acceptability of caesarean section may change (positively or negatively) as increasing numbers of women undergo the procedure, and that there is a need to understand how this relates to women’s perceptions of the quality of care. [52,42]

#### The value of interpersonal relationships during childbirth (SoFs 10)

In 13 studies [36,39,40,41,46,47,49,52–57,61,62] stakeholders reported valuing interpersonal relationships during labour and childbirth (including emotional labour, companionship and advocacy). In twelve high- and one middle-income country, women talked about their positive experiences of labour support from doulas and/or midwives. Health professionals also talked about the importance of partner support and one-to-one midwifery/nursing care in high-income settings where these were available. In middle-income settings the value of labour support was acknowledged, but availability was limited by too few midwives and inadequate facilities for partners to accompany women during labour.

#### Normative culture of intervention in childbirth (SoFs 11)

Stakeholder’s concerns that there was a normative culture of intervention in childbirth, and that this acted as an important barrier to caesarean section reduction, were voiced across high- and middle income settings. [36,39,42,46,47,49,50,52–57,61,62] These stakeholders were predominantly health professionals who valued medical care when used appropriately, but who also talked about how the over-medicalisation of childbirth may limit both their opportunities to fulfil their role optimally, and the opportunities for women to experience normal pregnancy and childbirth. Some health professionals, women, and managers perceived the advantages of vaginal birth to include increased speed of recovery, improved bonding between mother and child, shorter stays at the facility, lower costs for the health system, and, as stated by a decision-maker professional at a local level in Nicaragua, *“it is physiological*.*”* [55:p.2387] In contrast, there was recognition across settings, that “*some doctors’ routine prescription is intervention*.*”* [57:1377] That quote, from a participant in Iran, is illustrative of a general culture of intervention. Other stakeholders talked about specific practices, such as shift handover, where it was the norm for some staff engage in the process of “cleaning up”, about which, a paediatrician from the USA said: *“I’ll come in and the C-section fairy is on*.*”* [61:p.341]

#### Widely desired in principle but not universally acceptable in practice: standardising care (SoFs 12)

In 8 studies [40,42,50–55,57,58] health professionals and policy makers reported that shifts to standardise care were widely desired, but not universally acceptable in practice. Many stakeholders said they had high expectations of guidelines, care pathways or screening tools to reduce unnecessary caesarean section. They were particularly confident about such instruments of change if they were evidence based, designed to be used by multi-professional teams, and developed by consensus. However, discrepancies between what policy makers said existed and what health professionals said they were aware of were evident. [55] Participants from organisations with low caesarean section rates recognised that “*great effort has been made to ensure that staff are aware of national standards and guidelines*.*”* [40:p.45] Where intervention content imitated existing practices some health professionals welcomed them as legitimising and supportive of their clinical judgement, [50,52–54] while other staff in the same studies, particularly more experienced staff, experienced them as constraining of clinical judgement suggesting they encouraged *“robotic care”* through a *“tick-box-approach*.*”* [53:p.232] The burden of tools to audit and record standardised processes, and the time this took away from direct hands on care was also noted in one cross-country study. [58]

#### Attitudes towards in-practice use of best-evidence (SoFs 13)

One of the issues that underpinned the theoretical acceptance of standardised care, but the resistance to it in practice, was the notion of which standards are ‘good’ and how far population based evidence should always be used for individuals. In organisations with low caesarean section rates the normative culture was described as *“embracing of evidence and the drive to continually improve*.*” [*40:*p*.*45]* In organisations where new interventions were introduced with the aim to reduce caesarean section rates, without taking account of local health cultural norms, professionals reported how the underpinning evidence may be seen as credible or not depending on the prior beliefs and values of specific stakeholder groups. This is illustrated by a midwife in the UK who said “*It’s written down and because it’s coming from research*, *you’ve got all the references in front of you as to what type of research has been used and it sort of … just backs you up”*, while her obstetric colleague said of the same evidence “*We’re swapping one lot of vague-ish evidence for another lot of vague-ish evidence–and wait and see if anything goes wrong or not*”. [54:p.728] The selective use of evidence was reported by participants within studies, across resource settings. [42,47,50,54,55]

### Summary theme 3: Tackling too much caesarean section: Mechanisms of effect for change factors

The third summary theme comprises the components stakeholders identified as important to the implementation of interventions to reduce unnecessary caesarean section. This theme builds on the previous two, in illustrating some of the mechanisms to overcome entrenched power bases, and antagonistic cultural norms and behaviours.

#### Leading and following: Effective leadership, stakeholder involvement, and ownership to facilitate more positive attitudes towards changing workloads (SoFs 14 and 15)

In 14 studies from 13 countries, participants reported effective leadership, stakeholder involvement and ownership as crucial facilitators of commitment to reducing unnecessary caesarean section. [28,40,42,47,50–58,60–62] There was talk of the high priority caesarean section reduction should be given in the public domain (including media coverage) to engage women and their wider social networks. It was felt that this should be undertaken simultaneously with interventions across organisations, facilities and systems with respected, identifiable professional leaders at every level (both top-down and within and across peer-groups). The co-ordination of multiple mechanisms of commitment was considered essential to facilitating cultural and system change, because, as summed up by this manager, from the UK, “*if you want to implement something new*, *you need to get lots of stakeholders on board*.*”* [54:p.*727*] This also illustrates the important point that leaders can only lead effectively if they have followers who are convinced by their vision and the direction they are taking their organisation. Within and between studies, many participants expressed unmet needs for involvement in the development and implementation of interventions. For some professionals, opposition to change appeared to emerge from feelings of exclusion, alienation, limited sense of ownership, or lack of understanding of the underlying rationale for the change. [42,50,52–54,57,61] These factors were also observed in childbearing women, some of whom found it unacceptable that health professionals were making efforts to keep their labour physiological without understanding why. [52–52,61] The degree of opposition encountered was related to the extent to which an intervention was going against the local cultural norms. In such contexts, a lack of effective, sustainable leadership, little overt organisational buy-in, no mandatory requirement to change or no long-term accountability for caesarean section rates were associated with a lack of convinced followership, which was a significant barrier to change. As a midwife in Iran said “*One of the problems we have is that by presenting a program*, *we cannot expect the program to be implemented in the best way*.*”* [57: p1376] In another Iranian study, [47] and in Lebanon [42] and Chile [62], the need for a National Task Force with obstetric and midwifery representation was noted. Hospitals that achieved success in reducing rates identified nursing and medical leaders who endorsed and championed the project, and who made change an institution wide policy priority. [40,50,51,56]

Effective leadership, within and between professional groups, was also an important mediator of doctors and midwives’ openness to change in their everyday work. [42,46,47,49,50–58,60,61] This SoFs (15), is related to SoFs 7 (normative cultures of multi-disciplinary working between professionals) and others (including SoFs 3 and 4). It is distinct in its focus on attitudes towards the reassigning of workloads (shifting professional roles), new work (as a consequence of the intervention) and the importance of pre-existing workload pressures in implementation considerations. Across settings the importance of additional resource allocation was voiced. For example, in the UK, Japan, and Iran, midwives perceived midwifery care models as unmanageable unless more midwives were employed. [46,50,57] In Iran, it was also suggested that increasing the workloads of midwives had had the adverse effect of increasing caesarean section rates, as midwives came under pressure to free-up hospital beds. [47] Where interventions redefined the doctor’s role (family doctors and obstetricians) by shifting lead-professional responsibility to midwives, doctors discontent was evident. In the UK (Wales), doctors expressed concerns that they no longer had an overview of the overall maternity unit workload. Their new role, *“placed in a much more technical position”*, meaning they were confined to *“coming in like the fire brigade*.” [53:p.233;^54^:p732] Other doctors opposition to midwife led care was interpreted by study authors as fear of a shift in medical authority, loss of financial benefits, for both individuals and facilities, and the convenience of scheduled caesarean section, which made workloads more manageable (with less time on the wards, or on-call).

#### Addressing fears about safely reducing caesarean section rates through education and training (SoFs 16 and 17)

In 14 studies, stakeholder fears concerning the safely of reducing caesarean section rates were reported. [27,36,39,40,42,46,47,49,50,52–55,57,61,62] In the UK (in Scotland and Wales), fears about compromised clinical safety for women were described by doctors, and by some midwives, following a shift to midwifery-led models of care. [50,52–54] In contrast, in Canadian, UK and USA settings with the lowest caesarean section rates, midwives and obstetricians were more confident that support for women to give birth normally was where midwifery’s strength lay, with obstetric colleagues being well-trained to deal with any complications. [36,50,52–54,61] Practices and skill levels identified as facilitators of low caesarean section rates included “*well-trained*, *technically facile obstetricians who feel comfortable allowing a long 2*^*nd*^ *stage*, *who are competent at delivering breeches vaginally… and who encourage VBAC’s*.” [40: p,44] Despite this, while some decision-makers cited several advantages to vaginal birth, many health professionals focused on the risks. Defensive practice was talked about as a barrier to reducing unnecessary caesarean section in seven studies. [27,42,46,47,49,55,61] A lack of confidence in the safety of normal birth on the part of some women was also noted [27,52–54,61], with a Midwife in Iran suggesting one reason for this was that *“…society has spent more time on teaching the process of suing rather than introducing the labor to the general public*.” [47p:5].

The importance of education and training that prioritises normal birth and continuous quality improvement was reported in eight studies from high- and middle income settings. [40,42,46,47,55,56,57,61] The needs discussed included better prenatal education for women, and training of health professionals in clinical skills, clinical audit and the actual programme content of specific interventions or programmes targeted to reduce unnecessary caesarean section. The need for such training to be available and accessible to all stakeholders is encapsulated in this quote from a nurse in the US: *“I would provide the residents with more education on normal* … *I would want every single nurse on this unit to go through a childbirth education series*, *not the 1-day class*, *but a series*. *I would like to make the series available to every single patient here*, *at an affordable cost*. *Every single patient*!*”* [61;p342]

#### Dealing with complex adaptive systems by understanding, and tailoring to local context (SoFs 18 and 19)

The importance of understanding and effectively responding to local context, culture and pre-existing initiatives was evident in 16 studies as important mediators of negotiating support or resistance to change. [27,40,42,47–58,60–62] At country level distinctions were made between Chile and Lebanon for example. In Lebanon the convenience of caesarean section was suggested to be the foremost consideration with the need to address patience and skills in vaginal delivery in the “*new generation*” of obstetricians. Within countries there was also evidence of how the same interventions had different effects depending both upon the culture into which they were introduced and how they were accomplished therein. [50,52–54,56,62] Existing practice patterns, including maternal request for caesarean section, staff attitudes, relationships between professional groups and synergy with other initiatives (financial strategies and incentives, other guidelines and concurrent policies, evidence-based practice, local audit priorities) were all discussed. One UK study noted concurrent strategies intended to increase the normal birth rate (i.e. targeting home birth) as potential confounders, nevertheless caesarean section and instrumental delivery rates continued to rise, with the culture of individual units a significant factor. [53] There was recognition of the need for local tailoring of interventions, and for acknowledgment of how local culture must be actively and continuously negotiated as part of a wider system.

The subtleties of change-in-the-making were highlighted in 14 studies that reported how adaptive, multi-faceted interventions that accommodated local adaptation could optimally contribute to successful change programmes. [28,39,40,42,46,47,50–54,56,58–61] Examples of local adaptation included moving elective caesarean sections to a newly opened operating suite, which reduced scheduling conflicts that occurred when sharing space [51], obstetricians learning from midwives in ways they did not learn during their training about how to counsel women in early labour [61], and recognising “*obstetricians did not attend the initial meetings related to the initiative*”; but when “*a separate meeting was arranged to fit with their time commitments*”, that “*was well attended*.” [56: p337] Stakeholders described interventions that were continuously and creatively negotiated on-the-ground in ways that were not easily captured or anticipated. The mechanisms included inspiring confidence, and patience with variation in the length of time required to bring about change in different organisational cultural contexts. Some of the factors that contributed to development and effectiveness of interventions were opportunistic. For instance, they may have capitalised on other developments in other areas of the health system, so they were built alongside a general change in culture, rather than adherence to a particular checklist, or rigid protocol. Successful programmes also tended to have built-in mechanisms for multi-disciplinary collaboration and communication, and a commitment to continuous quality improvement so that adaptations could be made as evidence of local tinkering came to light. Without mechanisms to identify and address such issues, there was some evidence of no effect on caesarean section rates, or they continued to rise, as women previously identified as "normal" were re-classified as potentially "at risk" [52–54] or indications were found to fulfil insurance criteria [47].

**Fig 2:**
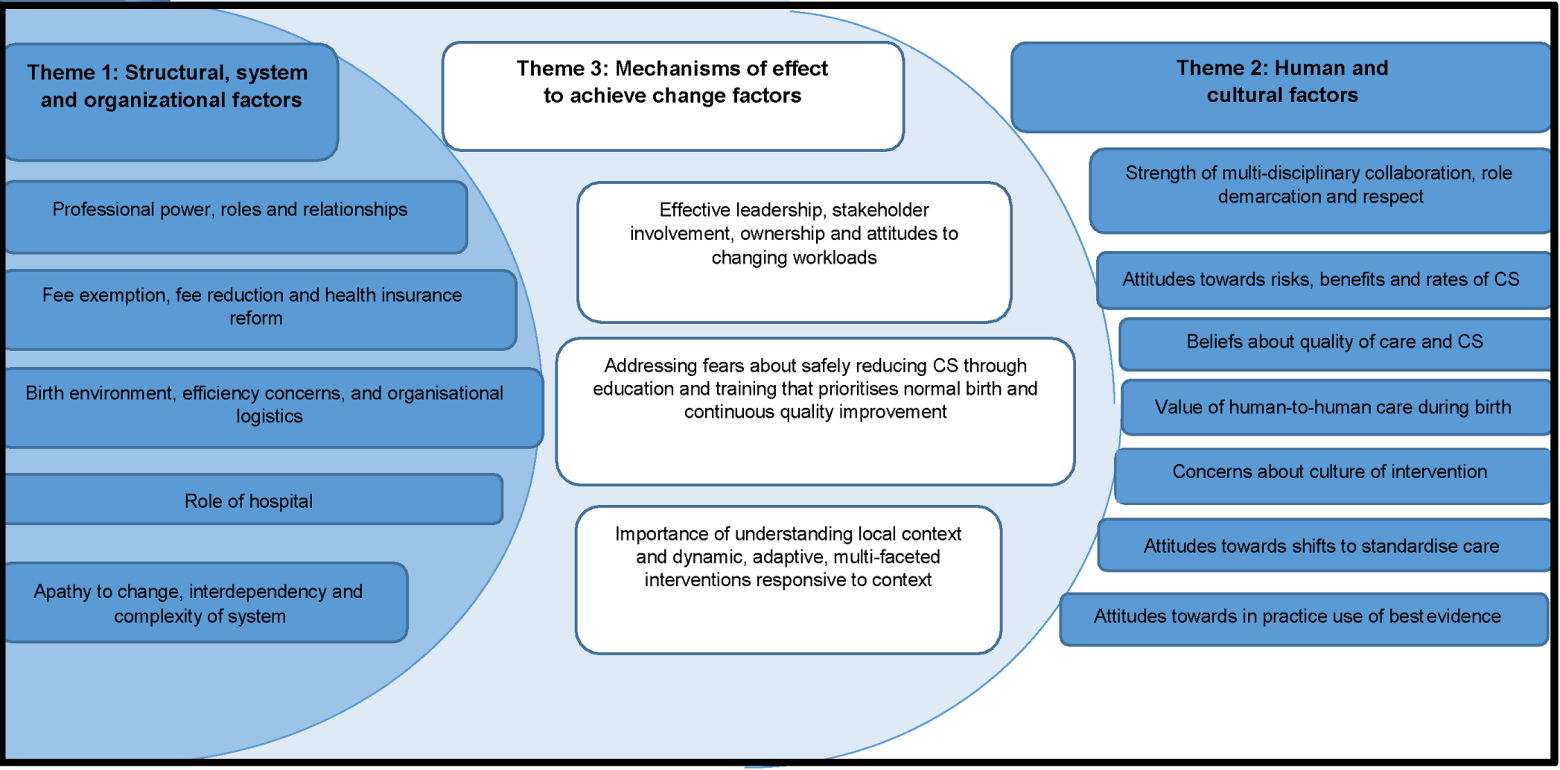
Summary of findings and summary themes.

In the final interpretive synthesis stage of the analysis (Fig 2) findings were combined to represent our interpretation, through a line of argument.

#### Line of argument synthesis

Maternity care is a complex adaptive system. Interventions to reduce caesarean section are unlikely to be successful unless account is taken of power, at all levels of the local health system and society, and until cultural norms and relationships are factored into the intervention process. Mechanisms of effect to achieve change include attention to effective leadership and followership; management of resistance to shifting power relations and to fear of responsibility for risk; and fostering of belief in the importance of reducing the caesarean section rate, with corresponding education of women and the training of health professionals. There is evidence to suggest this can be achieved by continuous dynamic assessment of, and tailoring to, local cultural norms and beliefs, as an essential and intrinsic part of the evaluation and implementation process of any new intervention or approach. Specific facilitators include multi-factorial programmes that build belief in, and valuing of, the need to reduce unnecessary caesarean section with all maternity stakeholders involved; authentic buy-in from effective leadership at all levels; three-way communication between women, midwives and doctors that includes listening as well as telling; and turning perceived losses (such as financial penalties, loss of professional roles and power, and perceived vulnerability to litigation) into gains (including pride in caesarean section rate, positive working relationships, better birth environments and improved quality of care for women and families).

## Discussion

Global health communities have begun to mobilise to address unnecessary caesarean section. [1–7,11,12,15–17] This systematic qualitative evidence synthesis illustrates how this societal willingness to change may not be effective or sustainable if it does not pay attention to the underlying mechanisms that incentivise or block successful social, organizational and system change. We found a combination of health system and cultural factors at play. This review makes explicit that approaches to optimize the use of caesarean section are more likely to succeed if they address stakeholders concerns about power, workloads and responsibilities; if they incorporate effective leadership and followership, and multidisciplinary teamwork, effective training (including women’s educational needs), collaboration and engagement; if they create a culture and environment that is consistent and supports policies, to ensure that system deficiencies do not create perverse incentives to increase caesarean section; if they consider and build upon stakeholders’ beliefs, fears and concerns on safety and quality of care; and if they have built-in adaptive mechanisms so that evolving is possible when unexpected local issues come to light.

Several quantitative systematic reviews, including a Cochrane Review, have previously evaluated the effectiveness and safety of interventions for reducing caesarean sections. [11,15–17,63–64] However, the interventions tested have resulted in limited success to date. The barriers and facilitators highlighted by this QES are a step forward to understanding why interventions may have limited success, how health system and cultural factors converge, and what the mechanisms of effect to achieve change are. It shows the interconnectedness between all stakeholders involved and how interventions to reduce unnecessary caesarean section ought to address the concerns and needs of each and every one. There is a reciprocal relationship between the design and delivery of health systems and organizations, the beliefs and values of service providers, and of service users, and the normative assumptions of local communities and societies. Each component of this interactive weave is shaped by the deficiencies, limitations and opportunities of local structures and cultures, and each has the potential to influence barriers and facilitators to change. Our findings provide a new point of departure for interventions in the future, that starts with understanding the mechanisms that are most likely to generate effective interventions, and that insists on local tailoring of the means of implementing these mechanisms, rather than with a one size fits all intervention.

### Limitations and strengths of the review

To the best of our knowledge this is the first global qualitative synthesis that brings together the evidence-base of what stakeholders say are the barriers and facilitators to the implementation of non-clinical interventions to reduce unnecessary caesarean section targeted at organizations, facilities and systems. Existing studies are sparse and limited, methodologically. We were unable to undertake the sub-analyses we planned, as there were too few studies in each sub-group to do this meaningfully. The systematic methodology and GRADE-CERQual assessment we used is a strength of the review, as is the inclusion of studies from 17 countries across high-, middle- and low-income settings, including three non-English language papers. [27–29]

### Implications for future research

Our findings suggest that some form of *a priori* formative research into a means of determining and accounting for local context and cultures may be of benefit in the design of multifaceted interventions in this area in the future, to ensure that likely mechanisms of effect are harnessed in the study design. Controlled studies of interventions, using adaptive designs, and including nested qualitative components that capture the nature and sustainability of local adaptation within randomised clusters of sites could add to the developing evidence base surrounding interventions to reduce unnecessary caesarean section. The use of the Robson’s 10 group classification is becoming increasingly internationally accepted as a means to monitor and compare caesarean section rates [7,65]. Routine monitoring of changes in practice may provide a foundation for best practice achievements that can be shared outside of traditional intervention randomised controlled trial designs. [2] The introduction of “living guidelines” provides an opportune platform to share best practice that can be emulated elsewhere. This may be more attuned to how the present review suggests change is achieved in practice.

### Conclusions

The global concern on the unprecedented increase of caesarean section has translated into societal willingness to change this trend by implementing interventions to optimize the use of caesarean section. This systematic review presents the evidence-based for critical structural, health system and organizational factors that will require careful local consideration in the design and implementation of such interventions. We propose that these factors are investigated in-depth in local initial formative research to ensure that likely mechanisms of effect are harnessed in the design of any intervention considered at country level.

## Supporting information

S1 Table PRISMA checklist. (DOCX)

S2 Table CERQual summary of evidence profile. (DOCX)

S1 Text Protocol. (PDF)

S2 Text Example search strategy. (DOCX)
