# Supplementary material for: Non-clinical interventions to reduce unnecessary caesarean section targeted at organisations, facilities and systems: Systematic review of qualitative studies
Source: PLoS One. 2018 Sep 4;13(9):e0203274. doi: 10.1371/journal.pone.0203274 (PMC6122831; doi:10.1371/journal.pone.0203274)
Supplement: S2 Table — (DOCX) [file pone.0203274.s002.docx]

**S2 Table 2, CERQual Summary of evidence profile**

| **Review finding** | **Contributing studies** |  |  |  |  | ***Assessment confidence in the evidence*** | ***Explanation of confidence in the evidence assessment*** |
| --- | --- | --- | --- | --- | --- | --- | --- |
| **Summary theme 1. Health system, organizational and structural factors** | | | | | | | |
| **Professional power, roles and relationships:** Where interventions challenged the balance of power between professionals, concerns within and between professional groups in practice were widespread. Stakeholders included obstetricians, midwives, family doctors and women. In organisations implementing MLC programmes there was dissatisfaction from doctors who felt their professional identity and the safety of women was compromised by relinquishing lead professional responsibility to midwives. There was some evidence that financial strategies to reduce costs for service users might enable midwives/local skilled birth attendants to refer women to facilities/obstetricians for CS more freely. | 42,46,47,49,50,52-54,57,58, 60,61,62 | No or minor concerns regarding methodological limitations with 4 studies of the highest quality and the rest high quality too | Minor concerns regarding coherence with data similar within and across studies. | Minor concerns about adequacy with thick data, from 5 geographical regions, 11 countries, 3 HICs, 3 MICs and 1 LIC. | Minor concerns about relevance with 9 of 11 studies highly relevant | High confidence | 11 studies with no or minor methodological limitations. Thick data from HICs and MICs with high CS rates. Thin data from LIC resource settings. High coherence. |
| **Fee exemption/reduction policies as mediators of access to necessary and unnecessary CS:** Across a number of studies, fee reduction policies were associated with a variable effect on appropriate use of CS dependent upon local philosophies of maternity care; inter-professional and inter-personal relationships; staff motivation to work with women or with the organisation, or simply for an income; and the expectations and demands of local women, families and communities. The unintended consequences of an increase in CS subsequent to reducing fees included longer-term iatrogenic damage to women’s health that is not covered by fee exemption. | 28,43,44,58,59,60 | Moderate concerns about methodological limitations with 2 very high quality studies, 3 moderate quality and 1 poor quality study | Moderate concerns regarding coherence with similar data within and across some studies | Moderate concerns about adequacy with some thick data. All studies from LICs were fee exemption or reduction policies exist | Moderate concerns about relevance with all included studies of medium relevance | Moderate confidence (in LIC and MIC settings where fee exemption or reduction polices exist) | 6 studies with no to major methodological limitations. All studies from LICs. Some thick data. Moderate coherence. |
| **Health insurance reform as a mediator of access to necessary and unnecessary CS:** Implementation of strategies to limit indications for CS accepted by insurance companies in Iran were met with scepticism about the power of insurance companies, concerns women who need a CS may no longer get one, and an increase in misreporting of indications for CS to satisfy amended insurance criteria. Insurance reform in China was not believed to be as influential on CS rates as women’s views of the advantages of CS. | 27,29,47, 48 | Moderate concerns about methodological limitations with 1 high quality study, 3 moderate quality studies | Major concerns regarding coherence with too few studies to assess across studies. | Major concerns about adequacy with 3 studies from China with thin data and 1 study from Iran with thick data. All studies from MICs. | Moderate concerns about relevance with one study highly relevant and 3 of medium relevance | Very low confidence | 4 studies with no to moderate methodological limitations. Major concerns about adequacy of data (thickness and spread). Too few studies contributed to this review finding to assess coherence. |
| **Birth environment, efficiency concerns and organisational logistics:** Only one included studied from the USA reported midwives’ views and experiences of birth in a home setting on the periphery (referring in if necessary) of birth in an organisation or facility, within a wider healthcare system. This study highlighted the absence of restrictions on women’s movements, environmental comforts, and time-limits evident in institutional settings. In the other studies contributing to this review finding a lack of time, space and facilities required for labour and normal birth were widely reported across resource contexts, as was access to operating theatres as a factor in clinical decision-making. In HICs where organisations had made changes to improve the birth environment and promote normal birth maintaining them was reported as a challenge (i.e. beds moved back in, resources for non-pharmacological forms of pain relief not prioritised). Insufficient space, insufficient staffing, lack of bathtubs, midwifery care not available for some women, and nutrition policies were commonly noted barriers. In MICs concerns were reported that delivery rooms were shared with other women (limiting presence of partner, family or other labour support companion), had inadequate facilities (lack of lighting, toilets, showers or baths, air-conditioning), or had been changed into operating theatres to accommodate rising numbers of CSs. | 28,36,39, 40-42,46, 47,49,  51,55-58,61,62 | No or minor concerns regarding methodological limitations in 13 of 16 studies and moderate concerns in 3 studies | Minor concerns regarding coherence with similar data within and across studies | Minor concerns about adequacy with thick data from across 5 geographical regions, HICs, MICs and LICs | Minor concerns about relevance with 14 of 16 included studies highly relevant. | High confidence | 16 studies, most with minor methodological limitations. Thick data from 5 geographical regions and all resource settings. High coherence. |
| **Role of hospital in acceptability of interventions to reduce unnecessary CS:** Type of hospital (public, private, university teaching, regional referral) and degree of autonomy over management were reported as important determinants of actual CS rates in organisation or facilities. The importance of relationships between hospitals and out-of-hospital care providers to facilitate referral in if needed was also noted. | 36,42-44, 46,47,55 | Minor to moderate concerns regarding methodological limitations | Moderate concerns regarding coherence with some data similar within and across studies, but few in number | Moderate concerns about adequacy with some thick data, included studies from across resource settings (HICs, MICs, and 1 LIC) but too few | Minor concerns about relevance with 5 of 7 studies highly relevant | Moderate confidence | 7 studies with no to moderate methodological limitations. Thick data from MICs. One LIC study. Moderate coherence. |
| **Apathy to change rooted in the interdependency of overall structure and complexity of healthcare systems:** Across the world, in HIC, MIC and LICs stakeholders’ reported resistance to change rooted in the belief that the reasons for caesarean section rates are a hugely complicated series of events, including both clinical and non-clinical factors. | 28,43,46, 47,52-55,57-60 | Minor concerns regarding methodological limitations in 8 studies and moderate concerns in 2 study | Moderate concerns regarding coherence with different facets of complexity reported in papers but this variation is explained by very different systems | Major concerns about adequacy with little thick data (from 1 HIC) and thin data from 2 MICs and 5 LICs. Ten countries in total (European, Americas, Eastern Mediterranean, Western Pacific, and African regions) | Moderate concerns about relevance with 5 studies highly relevant and 5 of medium relevance to reducing unnecessary CS | Low confidence | 10 studies with minor to moderate methodological limitations. Only thin data from across 4 geographical regions with only moderate coherence. |
| **Summary theme 2: Human and cultural factors** | | | | | | | |
| **Strength of multi-disciplinary collaboration, teamwork, communication, role demarcation and respect across maternity care system:** Policy makers and practising health professionals, across HIC and MICs reported effective teamwork as a key component to tackling unnecessary CS. Across setting organisations with the highest CS rates reported experiencing more challenges in achieving multi-disciplinary working within and between midwives and obstetricians, in organisational culture and in policy documents. | 40,42,46,47,49,50-58,60-62 | No or minor concerns regarding methodological limitations in 13 of 15 studies and moderate concerns in 2 studies | Minor concerns regarding coherence with similar data within and across studies | Minor concerns about adequacy with thicker data from HICs and MICs, but some from LICs. Data from across 5 geographical regions. | Minor concerns about relevance with 13 of 15 included studies highly relevant | High confidence | 15 studies, most with minor methodological limitations. Some very thick data from HICs and MICs. Data from all resource settings and 5 geographical regions. High coherence. |
| **Attitudes towards risks, benefits and organisational rates of CS:** In HIC and MICs health professionals had varying attitudes towards the value of CS. Some claimed a lack of awareness of any ill-effects of CS or their facility’s CS rate, others acknowledged their rates where high and risks existed but considered them “ignorable”, while some expressed specific concerns about anaesthetic risks, surgical complications, increased recovery time, cost longer term consequences for women. Women in Ghana were aware both that access to a health insurance scheme that gave them free maternity care could benefit them if they needed a CS, but also that this lead to an increase in CS rates and increased morbidity for some women. | 36,39,42,46,47,50-56,59,61 | No or minor concerns regarding methodological limitations in 11 of 12 studies and moderate concerns in 1 study | Minor concerns regarding coherence with similar data within and across studies, with variations explained by underpinning beliefs about CS or vaginal birth (HPs review) | Minor concerns about adequacy with some thick data from across 5 geographical regions, HICs, MICs and LICs | Minor concerns about relevance with 10 of 12 included studies highly relevant | High confidence | 12 studies with minor methodological limitations. Some thick data from across 5 geographical regions. High coherence. |
| **Belief quality of care for women is compromised or enhanced by reducing unnecessary CS:** In HIC and MICs inertia to change amongst some health professionals was rooted in perceptions of women’s preferences for obstetric-led care and CS. Some health professionals also perceived women as lacking in antenatal preparation for labour and vaginal birth. In the UK, US and Canada in organisations where care was actively focused on the promotion of normal birth health professionals reported positive impacts on women’s experience. | 27,39-42,46-55,57-62 | No or minor concerns regarding methodological limitations in 15 studies, and moderate concerns in 4 studies | Minor concerns regarding coherence with data similar within and across studies. Only variation in degree of concern | Minor concerns about adequacy with thick data, from 5 geographical regions, 15 countries, including LICs, but predominantly MIC and HICs | Minor concerns about relevance with 13 of 19 studies highly relevant | High confidence | 19 studies with minor methodological limitations. Thick data from 5 geographical regions. High coherence with variations in data explained by degree of concern. Studies predominantly from MICs and HICs with high CS rates. |
| **Valuing of human-to-human care during childbirth (including emotional labour, companionship and advocate for woman):** In HICs and one MIC women reported welcoming labour support from doulas or midwives. Health professionals talked about the importance of partner support and one-to-one midwifery/nursing care in HICs where these were available to many women. In MIC settings the value of labour support was recognised but availability was limited by too few midwives and inadequate facilities for partners to accompany women during labour. | 36,39,40,41,46,47, 49,52-57, 61,62 | No or minor concerns regarding methodological limitations in 11 studies, 3 moderate concerns | Moderate concerns regarding coherence with some data similar within and across studies, but also some variation, in part explained by negative attitudes of staff or unmet preferences of women. | Minor to moderate concerns about adequacy with thick data from 4 geographical regions, including HICs and MICs. No LICs. | Minor concerns about relevance with 11 of 13 studies highly relevant | Moderate confidence | 13 studies with no to moderate methodological limitations. Thick data from 4 geographical regions. Studies only from MICs and HICs. No LICs. Uncertain confidence in LICs. Moderate coherence. |
| **Concerns about culture of intervention in childbirth:** In HICs and MICs some stakeholders reported how the medicalization of childbirth can devalue it as a physiological process. Where interventionist organisational cultures were acknowledged as a problem, midwives and obstetricians talked about how it limited both their opportunities to fulfil their role optimally, and the opportunities for women to experience normal pregnancy and childbirth. | 36,39,42,46,47,49, 50,52-57, 61,62 | No or minor concerns regarding methodological limitations in 13 studies | Minor concerns regarding coherence with data similar within and across studies. Only variation in degree of concern | Minor concerns about adequacy with thick data, from 4 geographical regions, 8 countries, 5 MICs and 3 HICs. No LICs. | Minor concerns about relevance with 12 of 13 studies highly relevant | Moderate confidence | 13 studies with no or minor methodological limitations. Thick data from 4 geographical regions. High coherence. Studies only from MICs and HICs. No LICs. Uncertain confidence in LICs. |
| **Shifts to standardise care were widely desired but not universally acceptable in practice:** Across HICs and MICs many health professionals reported a desire for more standardised tools in the form of guidelines, care pathways, screening tools and audit. There were discrepancies between what policy makers said existed and clinicians said they were aware of. Where interventions were implemented they were variously received as legitimising existing good practice and supportive of clinical judgement; empowering for midwives faced with pressure from obstetricians against a shift from medical to midwifery-led care; or actively resisted, their formulation challenged (in terms of their evidence-base, or tick-box approach) and experienced as constraining of clinical judgement. The burden of tools (IT and other) to audit and record standardised processes, and the time this took away from direct hands on care, was also noted. | 40,42,50-55,57,58 | Minor concerns regarding methodological limitations in 7 studies and moderate concerns in 1 study | Moderate concerns regarding coherence with some variation in data across studies and settings, but explained by range of tools for standardisation and range of stakeholders | Moderate concerns about adequacy with some thick data from 2 of 4 studies from 2 HICs, 3 studies from 3 MICs and 1 study from 4 LICs with thin data | Minor concerns about relevance all included studies highly relevant | Moderate confidence | 8 studies with minor to significant methodological limitations. Very thin data from one study in LICs. High coherence. |
| **Attitudes towards in-practice use of best-evidence:** In HICs attitudes towards evidence varied. In some organisational cultures evidence was embraced as part of the drive for continuous quality improvement, whereas in others the quality of evidence underpinning programmes was questioned and/or organisations were selective in their use, particularly of evidence for midwifery-led care models. In MICs the desire for practice to be evidence-based was commonly discussed but felt to be not achievable in practice because of system limitations (resource, culture of intervention). | 40,42,47,50, 52-54,55 | No or minor concerns regarding methodological limitations in 5 studies, moderate concerns in 1 study | Moderate concerns regarding coherence with some similar data within and across studies, but variations but variations in how conceive what is evidence and how used | Moderate to major concerns in adequacy. Thin data, 3 studies from HICs (UK, Canada) and 3 MICs (Nicaragua, Iran and Lebanon) | Minor concerns about relevance with all studies highly relevant | Low confidence | 6 studies, most with no or minor methodological limitations. Data thin and only from HICs and MICs. Moderate coherence. |
| **Summary theme 3: Mechanisms of effect for change factors** | | | | | | | |
| **Effective leadership, stakeholder involvement and ownership:** Stakeholders reported the need for interventions to be publically given high priority across organisations, facilities and systems (including positive media coverage) with respected, identifiable leaders at every level (both top-down and within and across professional peer-groups) to make cultural change happen. All participants with a stake in maternity care (women, obstetricians, family doctors, midwives, policy makers, managers) reported the need for involvement in the development and implementation of interventions with opposition often stemming from feelings of exclusion, alienation and lack of ownership. Key considerations here were the degree of resistance encountered (see also local context) without effective, sustainable leadership, overt organisational buy-in, no mandatory requirement to change or long-term accountability for CS rates. Hospitals that achieved success in reducing rates identified nursing and medical leaders who endorsed and championed the project, made change an institution wide policy priority, not pilot or developmental. In a few MICs the need for a National Task Force with obstetric and midwifery representation was noted (Iran, Lebanon, Chile). | 28,40,42,47, 50-58,60-62 | No to minor concerns regarding methodological limitations in 11 studies and 3 moderate concerns | Minor concerns regarding coherence with similar data within and across studies | Minor concerns about adequacy with thick data across 4 geographical regions, 12 countries, and HIC, MIC and LICs resource settings | Minor concerns relevance with 11 studies highly relevant | High confidence | 14 studies with no to moderate methodological limitations. Thick data from 4 geographical regions and across resource settings. High coherence. |
| **Health professionals’ attitudes towards changing workloads:** Across the world, in all resource settings implementing interventions had consequences for everyday workloads. Insufficient resources for designated staff or dedicated time to work towards the successful implementation of interventions was viewed negatively the world over. In the UK MLC initiatives that made midwives the lead professional increased individual midwives workload (rather than putting more midwives in the system) and changed the nature of doctor’s workload by limiting their interpersonal involvement with women and making it harder for them to anticipate demand. In MICs increasing workloads of midwives to the point where they were stretched was reported to be a factor increasing CS rates, not reducing them as midwives came under intense pressure to free up beds. | 42,46,47,49, 50-58,60,61 | No or minor concerns regarding methodological limitations in 11 of 13 studies. Moderate concerns in 2. | Minor concerns regarding coherence with data similar within and across studies, where views were sought about actual interventions or development thereof | Minor concerns about adequacy with some thick data, from 5 geographical regions, 12 countries, across HIC, MIC and LIC resource settings | Minor concerns about relevance with 11 of 13 studies highly relevant | High confidence | 13 studies, most with no or minor methodological limitations. Thick data from across geographical regions and resource settings. High coherence. |
| **Fears about safely of reducing CS rates and skills and confidence to deliver normal birth amongst obstetricians, midwives and women:** In HICs and MICs some obstetricians and some midwives raised concerns about their professions competency to change and deliver more women vaginally, while in HIC settings with lower CS rates midwives and obstetricians were more confident that normal birth is where midwifery’s strength lies and obstetric colleagues were well-trained to deal with complications should they arise (i.e. high level surgical/operative skills, vaginal breech skills, and forceps skills). In MICs decision-makers cited several advantages to vaginal birth, while physicians focused on the disadvantages favouring CS to prevent any complications arising, particularly amongst women who live in isolated areas with little access to specialists should they need one. A lack of confidence in normal birth on the part of women was also noted. | 27,36,39,40,42,46, 47,49, 50,52-55, 57,61, 62 | No or minor concerns regarding methodological limitations in 13 of 14 studies | Minor concerns regarding coherence with data similar within and across studies, and variations explained by different resource settings and/or culture (interventionist or care) | Moderate concerns about adequacy with thick data, from 4 geographical regions, 10 countries, HICs and MICs. No LICs. | Minor concerns about relevance with 12 of 14 studies highly relevant | Moderate confidence | 14 studies with no or minor methodological limitations. Thick data from HICs and MICs. No data from LIC resource settings. High coherence. |
| **Education and training that prioritises normal birth and continuous quality improvement:** Various education needs in order to implement system change and reduce unnecessary caesarean section were identified by stakeholders. These included better prenatal education for women and better training of health professionals in clinical skills, clinical audit and the programme content of a specific interventions targeted to reduce unnecessary CS. | 40,42,46, 47, 55,56, 57,61 | Minor concerns regarding methodological limitations with 6 studies no or minor limitations and 1 moderate | Major concerns regarding coherence with wide variations in training needs identified by different stakeholders (concern some of which were perceptions of needs of others – women, midwives, junior doctors) | Moderate concerns about adequacy with thin data across 4 geographical regions, 6 countries, 4 studies from MICs, 3 from HICs. No LICs. | Minor concerns relevance with all studies highly relevant | Low confidence | 8 studies with minor to moderate methodological limitations. Thin data from 4 geographical regions. No LICs. Uncertain coherence. |
| **Importance of understanding local context, culture and existing initiatives that influence how favourable an organisation, facility or system is to reducing unnecessary CS:** Stakeholders views (policy makers, healthcare managers, health professionals and women) highlighted the importance of understanding local context in negotiating support and resistance to change. Understanding current practice patterns (including maternal request for CS), pre-existing initiatives (financial strategies and incentives, other guidelines, evidence-based practice, local audit priorities), and the importance of stakeholder involvement in the design of interventions were discussed with understanding where an organisation, facility or system is currently at as fundamental to the acceptability of an intervention. | 27,40,42, 47-58,60-62 | No or minor concerns regarding methodological limitations in 13 studies and moderate concerns in 3 studies | Minor concerns regarding coherence with similar data in and across studies | Minor concerns about adequacy with some thick data from 6 geographical regions, 12 countries and HIC, MIC and LIC settings | Minor concerns about relevance with 12 of 16 studies highly relevant | High confidence | 16 studies with minor methodological limitations. Thick data from 6 geographical regions, 12 countries and all resource settings. High coherence. |
| **Adaptive, multi-faceted interventions with local ‘tinkering’ acknowledged as components in success (or failure):** Stakeholders views and experiences of interventions show how they are not implemented in isolation. They are continuously and creatively negotiated on-the-ground in ways not easily captured or anticipated (administrator pride in revenue from increased CSs, length of time to bring about change different in different contexts). The factors that contributed to an interventions effectiveness were often opportunistic (i.e. capitalised on other developments in other areas of the health system) and reflected a change in culture, rather than adherence to a particular checklist or rigid protocol. They also had to have built-in mechanisms for multi-disciplinary collaboration and communication for continuous quality improvement that were adaptive to local ‘tinkering’ (i.e. women previously identified as "normal" classified as potentially "at risk", meaning the increased status of midwifery work was compromised by a reduced scope of practice in programmes for MLC or normal birth in HIC and MICs). | 28,39,40, 42,46,47, 50-54, 56,58-61 | Minor concerns regarding methodological limitations in 12 studies and moderate concerns in 2 studies. | Moderate concerns regarding coherence with similar data within and across studies at conceptual level, but variations in what is actually said and done | Moderate concerns about adequacy with thin data across 5 geographical regions, HICs, MICs and LICs | Minor to moderate concerns about relevance with 9 of 14 studies highly relevant | Moderate confidence | 14 studies with moderate to minor methodological limitations. Thin data from 5 geographical regions and all resource settings. Moderate coherence. |
