# Supplementary material for: Non-clinical interventions to reduce unnecessary caesarean section targeted at organisations, facilities and systems: Systematic review of qualitative studies
Source: PLoS One. 2018 Sep 4;13(9):e0203274. doi: 10.1371/journal.pone.0203274 (PMC6122831; doi:10.1371/journal.pone.0203274)
Supplement: S1 Text — (PDF) [file pone.0203274.s003.pdf]

The use of interventions to reduce unnecessary caesarean sections targeted at organizations, facilities and systems: a qualitative evidence synthesis

Carol Kingdon, Soo Downe, Ana Betrán

Citation

Carol Kingdon, Soo Downe, Ana Betrán. The use of interventions to reduce unnecessary caesarean sections targeted at organizations, facilities and systems: a qualitative evidence synthesis. PROSPERO 2017 CRD42017059456 Available from:
http://www.crd.york.ac.uk/PROSPERO/display_record.php?ID=CRD42017059456

Review question

This review focuses on interventions targeted at organizations, facilities, and systems to reduce unnecessary caesarean sections. The aim of this review is to add new evidence of implementation-related factors, including barriers and facilitators, feasibility, and meaningfulness.
The objectives of the review are to identify, appraise, and synthesize qualitative studies exploring:
1. What are stakeholders'* views of the different types of nurse/midwife and physician staffing interventions to reduce rates of unnecessary caesarean section? *We will not predefine stakeholders. We will post-define as anyone whose view has been sought on an intervention.
2. What are stakeholders' views and experiences of interventions to change the physical environment of labour to reduce unnecessary caesarean section rates?
3. What are stakeholders' views of interventions in which predetermined caesarean section rates are set at physician-, hospital- or regional-level?
4. What do stakeholders say about the barriers, facilitators, and ethical considerations of financial strategies to reduce unnecessary caesarean sections?
5. What are the views and experiences of stakeholders of the use of legal liability interventions for targeting reductions of unnecessary caesarean sections?
6. What do stakeholders say are the factors most important in organizational cultures committed to reducing unnecessary caesarean sections?

Searches

Electronic searches:
We will search the following electronic databases for eligible studies from 1985 to the date the final search is run:
• CINAHL (EBSCO);
• MEDLINE (EBSCO);
• PsycINFO (EBSCO);
• EMBASE (Ovid);
• Global Index Medicus;
• POPLINE;
• African Journals Online.
Using guidelines developed by the Cochrane Qualitative Research Methods Group for searching for qualitative evidence (Noyes 2011; Booth 2016), and papers detailing strategies for optimizing the identification of qualitative studies in CINAHL (Wilczynski 2007), MEDINE (Wong 2004), EMBASE (Walters 2006) and PsycINFO (McKibbon 2006), we will develop search strategies for each database. We chose these databases as we anticipated that they would provide the highest yield of results based on preliminary, exploratory searches. There will be no geographic restrictions imposed on the search, and the date restriction is intended to ensure that interventions implemented since the first WHO (1985) statement on appropriate technology for childbirth and use of caesarean section only when necessary are captured.
Searching other resources:

We will search the reference lists of all the included studies and key references (i.e. relevant systematic reviews), both back chaining and forward checking for any additional references not identified in the electronic searches that may be relevant. Key articles cited by multiple authors (citation pearls) will also be checked on Google Scholar, and the authors of relevant published protocols contacted.

Types of study to be included

This is a qualitative evidence synthesis, and as such, we will include all studies which have utilized qualitative designs (e.g. ethnography, phenomenology) or qualitative methods for data collection (e.g. focus group interviews, individual interviews, observation, diaries, oral histories), and which have used qualitative methods for data analysis (e.g. thematic analysis, framework approach, grounded theory, thematic network analysis).We will also include mixed methods studies if it is possible to extract findings derived from the qualitative research. We will exclude studies in which data has been collected using qualitative methods, but a qualitative analysis has not been conducted (for example, if qualitative data are only reported using descriptive statistics).

Condition or domain being studied

The following working definition of unnecessary caesarean section will be used for the purposes of this review:
'Unnecessary caesarean deliveries are those procedures that are performed in the absence of medical indications such as substantial maternal risk factors, fetal anomalies, pregnancy complications, birth weight < 2500 g or > 4000 g, and complications of labour or delivery (Koroukian 1998). Generally unnecessary caesarean deliveries are those without medical indications in which the mother is exposed to potential harms that outweigh the potential benefits (Kabir 2004).'

Participants/population

We will include studies which have focussed on aggregate-level interventions to reduce unnecessary caesarean sections targeted at organizations, facilities and healthcare systems. This means that the types of participants may include:
• Policy makers;
• Healthcare managers;
• Healthcare professionals;
• Anyone else charged with operationalizing an intervention at an organizational, facility, healthcare system or societal level.

Intervention(s), exposure(s)

This review is focused on interventions to reduce unnecessary caesarean sections targeted at organizations, facilities and healthcare systems. We define an intervention as 'anything considered by the study authors as an intervention additional to usual care undertaken with the aim of reducing unnecessary caesarean section.' Of particular interest are aggregate-level (high-level) interventions in society (i.e. legislative change) and healthcare (i.e. policy change) interventions.
Inclusion criteria:
We will include qualitative or mixed-method studies about interventions conveyed through any medium which explore the values and preferences of individuals and groups charged with operationalizing interventions in organizations, facilities, healthcare systems and society. These interventions will include:
• Interventions aimed at changing organizational culture;
• Insurance reforms;
• External peer review;
• Legislative policy limiting financial/legal liability in case of litigation;
• Legislative policy regarding women's reproductive rights;
• Staffing models;
• Specific goals for caesarean section rates;
• Targeted financial strategies.
Exclusion criteria:
We will not include interventions targeted at women, communities or the public, or at health professionals, as

these interventions are the subject of two other ongoing reviews.

Comparator(s)/control

Not applicable.

Primary outcome(s)

Studies have shown that health system factors are important aggregate-level determinants of caesarean section use (Lauer 2010). We also know that healthcare systems function as complex microcosms of the society of which they are part, within which social change, legislation, the effects of healthcare financing, and the threat of litigation all impact caesarean section rates across time and place. Less is known, however, about how aggregate-level interventions to reduce unnecessary casarean sections are received in society, and the human factors which influence their successes or failures. This review will provide that evidence.

Secondary outcome(s)

Not applicable.

Data extraction (selection and coding)

We will collate records identified from different sources into one database and will remove duplicates. Two review authors (CK, SD) will independently assess each abstract in order to determine inclusion against the a priori eligibility criteria. At this stage we will disregard those abstracts which are clearly irrelevant to the topic of this review. The same two review authors (CK, SD) will then retrieve the full texts of all the papers which are likely to be relevant, and will independently assess them, before agreeing on the final list of included studies. In the event of any continuing lack of agreement over a particular study, a third review author (AB) will adjudicate, and if appropriate, we will contact the study authors for further information.
We will record study characteristics using a form designed specifically for this review. The form will record details of: first study author, date of publication, language, country of study, setting (public, private), context (urban/rural), region (African, Americas, South-East Asian, European, Eastern Mediterranean, Western Pacific), participant group (parity, socio-demographics), the type of intervention received, the theoretical/conceptual perspective of the study, research methods, sample size, method of analysis, and the key themes (as recorded by the study authors in each case).

Risk of bias (quality) assessment

Our inclusion criteria specify that in order to be included in the review, a study must have used qualitative methods for both data collection and data analysis, and these must be described in the paper. This criterion constitutes a basic quality threshold, as studies which do not meet this standard will be discarded. In addition, in order to assess the methodological quality of included studies, one review author will apply a quality appraisal framework to each study, and a second review author will check for discrepancies.
Disagreements will be resolved through discussion, or by consulting a third review author. We will use the criteria from Walsh (2006) and the A-D grading of Downe (2007) which includes an assessment of the study scope and purpose, design, sampling strategy, analysis, interpretation, researcher reflexivity, ethical dimensions, relevance, and transferability. We will then grade studies against Lincoln and Guba’s summary criteria (Lincoln 1985), as follows:

- A: No, or few flaws. The study credibility, transferability, dependability, and confirmability is high.
- B: Some flaws, unlikely to affect the credibility, transferability, dependability, and/or confirmability of the study.
- C: Some flaws that may affect the credibility, transferability, dependability, and/or confirmability of the study.
- D: Significant flaws that are very likely to affect the credibility, transferability, dependability, and/or confirmability of the study.

Two review authors will independently conduct a pilot on three included studies to assess the feasibility of using this tool, and to evaluate the integrity of the assessment, which will be agreed by consensus. As previously stated, studies meeting the inclusion criteria will be included regardless of study quality. Quality assessment scores will be used when judging the relative contribution of each study in the development of explanations and relationships between studies, with the synthesis becoming “weighted” towards the

findings of the better quality studies (Glenton 2013).
We will use the GRADE Confidence in the Evidence from Reviews of Qualitative research (GRADE-CERQual) approach to assess the confidence that may be placed in review findings (Lewin 2015) by applying the following four domains:
• Methodological limitations of included studies: the extent to which there are problems in the design or conduct of the primary studies that contributed evidence to a review finding.
• Relevance of the included studies to the review question: the extent to which the body of evidence from the primary studies supporting a review finding is applicable to the context (perspective or population, phenomenon of interest, setting) specified in the review question.
• Coherence of the review finding: the extent to which the review finding is well grounded in data from the contributing primary studies and provides a convincing explanation for the patterns found in these data.
• Adequacy of the data contributing to a review finding: an overall determination of the degree of richness and quantity of data supporting a review finding.

Strategy for data synthesis
Following the principles of meta-ethnography (Noblit and Hare 1988), we will undertake data extraction and analysis simultaneously. Meta-ethnography uses an approach based on the constant comparative technique, in which the analysis is built up study by study using the principles of confirmation ('reciprocal analysis') and dis-confirmation ('refutational analysis'). Starting with the earliest published paper, we will read each included study in detail, and will extract the relevant verbatim text, along with the themes/theories/metaphors used by the study authors. Two review authors (CK, SD) will undertake the analysis, and any disagreements on the thematic structure/theory/amendments will be agreed by consensus throughout the extraction and analysis process. We will synthesize the resultant thematic structure into a ‘line of argument’ synthesis, before assessing the degree of confidence that can be placed in the evidence from the review findings (CERQual).

Analysis of subgroups or subsets
Our data management and synthesis plan is intended to support the following sub-analysis:
Data from low- and middle-income countries, and those from high-income countries.
We propose this sub-analysis due to differences in uptake, health beliefs, and health system accessibility and quality between these two types of settings.

Contact details for further information
Carol Kingdon
ckingdon@uclan.ac.uk

Organisational affiliation of the review
World Health Organization

Review team members and their organisational affiliations
Dr Carol Kingdon. University of Central Lancashire
Professor Soo Downe. University of Central Lancashire
Dr Ana Betrán. World Health Organization

Anticipated or actual start date
03 January 2017

Anticipated completion date
28 July 2017

Funding sources/sponsors
World Health Organization

Conflicts of interest
None known

National Institute for Health Research

Language
English

Country
England

Stage of review
Review_Ongoing

Subject index terms status
Subject indexing assigned by CRD

Subject index terms
Cesarean Section; Delivery of Health Care; Female; Health Care Facilities, Manpower, and Services; Humans; Organizations; Parturition; Pregnancy; Pregnancy Outcome; Unnecessary Procedures

Date of registration in PROSPERO
18 May 2017

Date of publication of this version
18 May 2017

Details of any existing review of the same topic by the same authors

Stage of review at time of this submission

Stage	Started	Completed
Preliminary searches	Yes	Yes
Piloting of the study selection process	Yes	No
Formal screening of search results against eligibility criteria	No	No
Data extraction	No	No
Risk of bias (quality) assessment	No	No
Data analysis	No	No

Versions
18 May 2017

PROSPERO
This information has been provided by the named contact for this review. CRD has accepted this information in good faith and registered the review in PROSPERO. CRD bears no responsibility or liability for the content of this registration record, any associated files or external websites.
